# Timing of Red Blood Cell Transfusions and Occurrence of Necrotizing Enterocolitis: A Secondary Analysis of a Randomized Clinical Trial

**DOI:** 10.1001/jamanetworkopen.2024.9643

**Published:** 2024-05-03

**Authors:** Ariel A. Salas, Elizabeth Gunn, Waldemar A. Carlo, Edward F. Bell, Abhik Das, Cassandra D. Josephson, Ravi M. Patel, Sylvia Tan, Haresh Kirpalani

**Affiliations:** 1 Department of Pediatrics, University of Alabama at Birmingham; 2 Department of Pediatrics, University of Iowa, Iowa City; 3 Statistical and Environmental Sciences Unit, RTI International, Washington, DC; 4 Cancer and Blood Disorders Institute and Blood Bank/Transfusion Medicine, Johns Hopkins All Children’s Hospital, St Petersburg, Florida; 5 Department of Oncology, John Hopkins University School of Medicine, Baltimore, Maryland; 6 Department of Pediatrics, Emory University, Atlanta, Georgia; 7 Department of Pediatrics, University of Pennsylvania, Philadelphia

## Abstract

**Importance:**

Observational studies often report that anemia and red blood cell (RBC) transfusions are associated with a higher risk of necrotizing enterocolitis (NEC) among extremely low-birthweight (ELBW) infants.

**Objective:**

To evaluate whether there is a temporal association between 72-hour hazard periods of exposure to RBC transfusions and NEC among ELBW infants randomized to either higher or lower hemoglobin transfusion thresholds.

**Design, Setting, and Participants:**

This post hoc secondary analysis of 1690 ELBW infants who survived to postnatal day 10 enrolled in the Transfusion of Prematures (TOP) randomized multicenter trial between December 1, 2012, and April 12, 2017, was performed between June 2021 and July 2023.

**Exposures:**

First, the distribution of RBC transfusions and the occurrence of NEC up to postnatal day 60 were examined. Second, 72-hour posttransfusion periods were categorized as hazard periods and the pretransfusion periods of variable duration as control periods. Then, the risk of NEC in posttransfusion hazard periods was compared with that in pretransfusion control periods, stratifying the risk based on randomization group (higher or lower hemoglobin transfusion threshold group).

**Main Outcomes and Measures:**

The primary outcome was incidence of NEC stage 2 or 3. Secondary outcomes included the incidence rates of NEC within five 10-day intervals, taking into account the number of days at risk.

**Results:**

Of 1824 ELBW infants randomized during the TOP trial, 1690 were included in the present analysis (mean [SD] gestational age, 26.0 [1.5] weeks; 899 infants [53.2%] were female). After categorizing 4947 hazard periods and 5813 control periods, we identified 133 NEC cases. Fifty-nine of these cases (44.4%) occurred during hazard periods. Baseline and clinical characteristics of infants with NEC during hazard periods did not differ from those of infants with NEC during control periods. The risk of NEC was 11.9 per 1000 posttransfusion hazard periods and 12.7 per 1000 control periods (adjusted risk ratio, 0.95; 95% CI, 0.68-1.32; *P* = .74). This risk did not differ significantly between randomization groups, but the incidence rate of NEC per 1000 days peaked between postnatal days 20 and 29 in the lower hemoglobin transfusion threshold group.

**Conclusions and Relevance:**

The findings of this post hoc analysis suggest that, among ELBW infants with the hemoglobin ranges occurring in the TOP trial, exposure to RBC transfusions was not temporally associated with a higher risk of NEC during 72-hour posttransfusion hazard periods. Given that the incidence rate of NEC peaked between postnatal days 20 and 29 among infants with lower hemoglobin values, a more in-depth examination of this at-risk period using larger data sets is warranted.

**Trial Registration:**

ClinicalTrials.gov Identifier: NCT01702805

## Key Points

**Question:**

Are hazard periods defined by exposure to red blood cell (RBC) transfusions associated with a higher risk of necrotizing enterocolitis (NEC) among extremely low-birthweight (ELBW) infants?

**Findings:**

In this secondary analysis of the Transfusion of Prematures (TOP) randomized clinical trial, 1690 ELBW infants experienced 4947 hazard periods of exposure to RBC transfusions and 5813 control periods of nonexposure. With a total of 133 cases of NEC, the frequency of NEC did not differ significantly between posttransfusion hazard periods and pretransfusion control periods (11.9 vs 12.7 per 1000 periods, respectively).

**Meaning:**

This study’s findings suggest that, when compared with control periods, 72-hour hazard periods after exposure to RBC transfusions are not temporally associated with a higher risk of NEC among ELBW infants with the hemoglobin ranges outlined by the TOP trial.

## Teaser

This secondary analysis of a randomized clinical trial evaluates the temporal association between 72-hour hazard periods of exposure to red blood cell (RBC) transfusions and necrotizing enterocolitis among extremely low-birthweight infants randomized to higher or lower hemoglobin transfusion thresholds.

## Introduction

Observational and preclinical studies, using a diverse array of analytic approaches, often report that both anemia and red blood cell (RBC) transfusions are associated with a higher risk of necrotizing enterocolitis (NEC),^1,2,3,4,5,6,7^ a gastrointestinal disease that accounts for 10% of neonatal deaths and increases the risk of severe morbidity among survivors.^8,9^ However, numerous randomized clinical trials have shown that giving more RBC transfusions to extremely low-birthweight (ELBW) infants randomized to higher hemoglobin transfusion thresholds is not associated with a higher risk of NEC.^10,11,12,13,14^ For example, the largest randomized clinical trial comparing higher vs lower transfusion thresholds, the Transfusion of Prematures (TOP) trial, recruited 1824 ELBW infants, maintained hemoglobin values greater than or equal to 7 g/dL (to convert to grams per liter, multiply by 10.0), and reported no significant differences in the secondary outcome of NEC (relative risk, 0.95; 95% CI, 0.73-1.25).^10^ These trials did not examine temporality of NEC after an RBC transfusion, leaving this question unanswered.

Our objective was to compare the risk of NEC during hazard periods of exposure to RBC transfusion with the risk of NEC during control periods of nonexposure among an at-risk cohort of ELBW infants who participated in the TOP trial. By focusing only on the NEC cases that developed shortly after exposure to RBC transfusions, we differentiated NEC associated with transfusions from NEC explained by other causes. We hypothesized that if a temporal association between RBC transfusions and NEC exists, then the risk of NEC would be transiently higher during hazard periods.

## Methods

### Study Design, Setting, and Participants

We performed a secondary analysis of the TOP trial (ClinicalTrials.gov: NCT01702805) (the full trial protocol is available in Supplement 1). The trial involved 19 centers participating in the Eunice Kennedy Shriver National Institute of Child Health and Human Development Neonatal Research Network.^10^ Infants born between December 1, 2012, and April 12, 2017, who had a birth weight of 1000 g or less, a gestational age between 22 weeks 0 days and 28 weeks 6 days, and a postnatal age of 48 hours or less were eligible for the trial. After written informed parental consent, infants were randomized to either a higher or lower hemoglobin transfusion threshold group (hereafter, higher or lower threshold group) in a 1:1 ratio within center and birth weight strata (<750 g vs ≥750 g). All sites received institutional review board approval for the study.^10^ The report of this post hoc secondary analysis of a prespecified trial outcome follows the Consolidated Standards of Reporting Trials (CONSORT) reporting guideline for randomized studies.

### Observation Period

**Figure 1.**
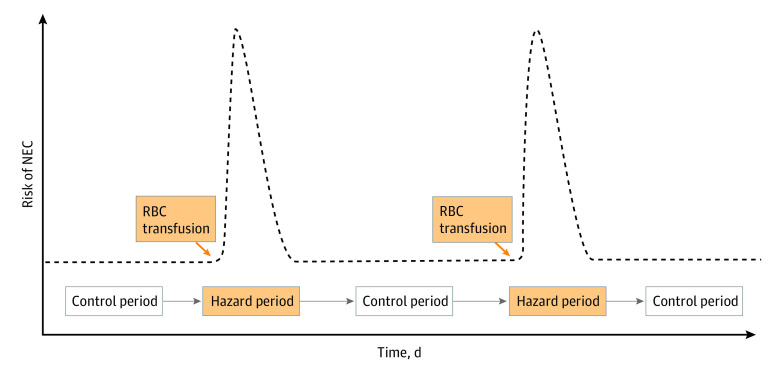
Conceptual Model If an association between red blood cell (RBC) transfusions and necrotizing enterocolitis (NEC) exists, the risk of NEC would be transiently high after a transfusion and low at baseline before a transfusion. The transient, high-risk, posttransfusion periods are considered hazard periods, and the low-risk, pretransfusion periods are considered control periods.

The conceptual model depicted in Figure 1 was used to establish temporality in the association between RBC transfusions and NEC. For instance, an infant undergoing a mean of 5 transfusions within a 50-day window from postnatal day 10 to 60 would contribute to the data set with 5 posttransfusion hazard periods and 5 pretransfusion control periods. The hypothesis was that the occurrence of NEC as a single primary outcome would be more common during posttransfusion hazard periods.

All infants who survived to postnatal day 10 were included. The observation period in this cohort began on postnatal day 10 and continued until postnatal day 60 or until the occurrence of NEC or death, if earlier. We chose this postnatal age range to remove potential confounding effects of acute critical illness in the first 10 days after birth and to minimize possible overlap with spontaneous intestinal perforation.^15^ By limiting the observation period to postnatal day 60, we excluded “late” cases of NEC, which are not reported to be associated with RBC transfusions.^5,16^

### Hazard or Exposure Periods

A hazard period (or the time after exposure to RBC transfusion with the highest risk of NEC) began on the hour of the transfusion and ended after 72 hours or at NEC diagnosis, if earlier. Hazard periods were shorter if an RBC transfusion occurred at the 2 ends of the observation period. For instance, at postnatal day 10, if an infant had received an RBC transfusion on postnatal day 8, the first 48 hours of the hazard period that began on postnatal day 8 were not included. Similarly, at the end of the observation period, if an infant received an RBC transfusion on postnatal day 58, only the first 48 hours of the hazard period were included. Hazard periods were longer if multiple RBC transfusions were given to the same infant less than 72 hours apart. In this case, hazard periods were consolidated into a single longer hazard period, which began on the hour of the first transfusion and ended 72 hours after the last transfusion (or at NEC diagnosis if earlier).

### Control or Nonexposure Periods

A control period was a nonhazard period between postnatal day 10 and 60. The duration of a control period was variable by definition. The maximum of 1200 hours (50 days) would correspond to study participants who did not receive any transfusions during the observation period.

### Primary Outcome

The primary outcome was NEC stage 2 or 3,^17^ as defined by radiographic appearance or surgery. In the TOP trial, NEC cases associated with RBC transfusions were treated as adverse events, for which clinicians would come to a consensus about the occurrence of NEC as a result of an RBC transfusion before reporting it. In addition, for this secondary study, the time of diagnosis was confirmed for all NEC cases. This allowed reassurance that clinical signs of NEC were not present before RBC transfusions. The study protocol did not provide specific recommendations for the ordering of abdominal radiographs throughout the trial. Instead, the decision to order abdominal radiographs for diagnosing NEC was left to the discretion of the clinicians. These radiographs were subsequently interpreted by radiologists as part of usual care. No deliberate attempts were made to standardize the timing for ordering abdominal radiographs based on clinical symptoms, nor was there a defined protocol for routinely obtaining additional radiographs shortly after a transfusion.

### Statistical Analysis

Statistical analysis was performed between June 2021 and July 2023. The primary objective of this study was to compare the risk of NEC during the defined 72-hour hazard periods with that during the control periods, both overall and as stratified by randomization group (higher or lower threshold group). Using Poisson regression, we adjusted for study site and for the correlation within patients. The stratified model tested the moderating association of randomization group with the risk of NEC for hazard vs control periods. To ensure that the length of our chosen hazard period had not introduced associations with the outcome, we performed a prespecified sensitivity analysis with a longer hazard period (120 hours). Last, to account for potential variation in the risk of NEC throughout the postnatal observation period, the primary analysis was repeated within 5 observation intervals: 10 to 19, 20 to 29, 30 to 39, 40 to 49, and 50 to 60 postnatal days. In addition, secondary analyses were performed to assess the incidence rates of NEC. These secondary analyses took into account the number of days at risk rather than periods at risk within each of the 5 intervals.

Clinical characteristics of infants who received a diagnosis of NEC during hazard and control periods were compared using the Fisher exact test for categorical variables and unpaired, 2-tailed *t* tests for continuous variables. A 2-sided *P* value of <.05 was considered significant. All statistical analyses were conducted using SAS software, version 9.4 (SAS Institute Inc).

The TOP trial reported that 186 ELBW infants developed NEC stage 2 or 3 during the trial. Because each TOP participant received a mean of 5 RBC transfusions, we estimated that 9130 hazard and control periods would be available for analysis. These estimates suggest that the rate of NEC during either a hazard or control period was approximately 20 per 1000 periods. With this fixed sample size, we determined that the statistical power to detect a difference of 5 per 1000 periods between hazard and control periods was approximately 67%, with a 2-sided α of .05.

## Results

Among the 1690 ELBW infants considered at risk of NEC after postnatal day 10 (eFigure 1 in Supplement 2), we identified 4947 hazard periods and 5813 control periods. The mean (SD) gestational age was 26.0 (1.5) weeks and the mean (SD) birthweight was 765 (147) g; 899 infants (53.2%) were female and 791 (46.8%) were male. A total of 85.1% of all control periods (4947 of 5813) were pretransfusion periods. The remaining 14.9% (866 of 5813) consisted of either isolated single control periods or control periods that began 72 hours after transfusion and ended by postnatal day 60. Hazard periods ran for a mean (SD) of 72.7 (27.5) hours (range, 0.03-511 hours). Control periods ran for a mean (SD) of 256.1 (254.7) hours (range, 0.02-1200 hours).

A total of 133 ELBW infants (7.9%) developed NEC stage 2 or 3 between postnatal day 10 and 60 (Table 1). Fifty-nine of these events (44.4%) occurred during hazard periods. Seventy-four events (55.6%) occurred during control periods (eFigure 2 in Supplement 2). Thirteen additional NEC events occurred after the observation period and 108 deaths occurred during the observation period.

**Table 1.** Comparison of Infants With NEC Diagnosed During Hazard vs Control Periods

Characteristic	No./total No. (%)			
	Overall (N = 133)	Diagnosed during hazard period (n = 59)	Diagnosed during control period (n = 74)	*P* value^a^
Maternal age, mean (SD), y	27.3 (6.1)	28.0 (6.4)	26.8 (5.9)	.29
Black race	70/131 (53.4)	34/57 (59.6)	36/74 (48.6)	.04
Married	42/133 (31.6)	19/59 (32.2)	23/74 (31.1)	>.99
Education past high school	42/112 (37.5)	19/52 (36.5)	23/60 (38.3)	.97
Public insurance	104/133 (78.2)	46/59 (78.0)	58/74 (78.4)	>.99
Multiple gestations	27/133 (20.3)	9/59 (15.3)	18/74 (24.3)	.28
Maternal hypertension	40/133 (30.1)	13/59 (22.0)	27/74 (36.5)	.09
Received antenatal corticosteroids	121/133 (91.0)	55/59 (93.2)	66/74 (89.2)	.55
Birth weight, mean (SD), g	754.6 (137.0)	744.4 (137.9)	762.7 (136.7)	.45
Gestational age, mean (SD), wk	25.8 (1.5)	25.6 (1.2)	26.0 (1.7)	.11
Infant sex				
Female	72/133 (54.1)	28/59 (47.5)	44/74 (59.5)	.22
Male	61/133 (45.9)	31/59 (52.5)	30/74 (40.5)	
Small for gestational age	11/133 (8.3)	5/59 (8.5)	6/74 (8.1)	>.99
Nutritional characteristics				
Age when birth weight was regained, mean (SD), wk	2.1 (1.0)	2.2 (1.0)	1.9 (1.0)	.16
No. of infants	130	58	72	
Duration of parenteral nutrition, mean (SD), d	45.7 (31.6)	41.0 (25.6)	49.5 (35.3)	.11
Age at first enteral feeding (DOL), mean (SD), d	4.2 (5.1)	3.6 (1.7)	4.7 (6.7)	.18
Age at full enteral feeding (DOL), mean (SD), d	25.6 (18.1)	25.9 (19.3)	25.3 (17.1)	.87
No. of infants	114	52	62	
Breast milk use in first 28 d, mean (SD), d	17.5 (6.9)	18.3 (7.6)	16.8 (6.3)	.31
No. of infants	94	45	49	
SNAP score, mean (SD)	21.4 (14.3)	21.5 (14.1)	21.3 (14.5)	.93
No. of infants	130	59	71	
SNAPPE score, mean (SD)	44.8 (21.6)	44.2 (22.4)	45.3 (21.1)	.76
No. of infants	128	58	70	
Transfusion characteristics				
No. of transfusions per infant, mean (SD)	6.9 (4.5)	7.7 (4.3)	6.3 (4.6)	.08
No. of protocol-triggered transfusions per infant, mean (SD)	5.3 (3.7)	5.8 (3.5)	4.9 (3.9)	.17
No. of extra clinical transfusions per infant, mean (SD)	1.6 (2.5)	1.9 (2.8)	1.4 (2.3)	.30
No. of transfusions during the first 10 DOL per infant, mean (SD)	1.6 (1.4)	1.7 (1.4)	1.5 (1.4)	.35

Abbreviations: DOL, days of life; NEC, necrotizing enterocolitis; SNAP, Score for Neonatal Acute Physiology; SNAPPE, Score for Neonatal Acute Physiology with Perinatal Extension.

^a^ From unpaired, 2-tailed *t* test for continuous variables and Fisher exact test for categorical variables.

### Primary Analyses Taking Into Account Periods at Risk

The risk of NEC was 11.9 per 1000 posttransfusion hazard periods and 12.7 per 1000 control periods (adjusted risk ratio, 0.95; 95% CI, 0.68-1.32; *P* = .74). The stratified analysis according to TOP randomization groups is shown in Table 2. The unadjusted risk ratio for developing NEC among hazard periods as compared with control periods was 0.91 (95% CI, 0.57-1.48) for the higher threshold group (*P* = .71) and 0.99 (95% CI, 0.62-1.58) for the lower threshold group (*P* = .97); these risk ratios were not different from one another (*P* = .81), indicating no apparent moderation of the risk of NEC by threshold group. Results from a sensitivity analysis with a 120-hour definition of hazard periods was not measurably different from the analyses with a 72-hour definition (eTable in Supplement 2).

**Table 2.** Stratified Analysis According to Randomization Groups^a^

Characteristic	Hemoglobin transfusion threshold group and period			
	Higher		Lower	
	Hazard periods (mean duration, 3 d)	Control periods (mean duration, 9 d)	Hazard periods (mean duration, 3 d)	Control periods (mean duration, 12 d)
No. of days	8659	29 621	6331	32 399
NEC events	29	36	30	38
No. of periods	2858	3230	2089	2583
NEC rate per 1000 periods	10.2	11.2	14.4	14.7

Abbreviation: NEC, necrotizing enterocolitis.

^a^ The unadjusted risk ratio for developing NEC among hazard periods vs control periods was 0.91 (95% CI, 0.57-1.48) for the higher hemoglobin transfusion threshold group and 0.99 (95% CI, 0.62-1.58) for the lower hemoglobin transfusion threshold group. These risk ratios were not different from one another; the ratio of risk ratios was 0.92 (95% CI, 0.47-1.80) (*P* = .81).

Dividing the follow-up period into 10-day intervals showed that the risk of NEC was highest between postnatal days 20 and 39. During postnatal days 20 to 29, the risk of developing NEC was significantly higher among hazard periods compared with control periods (adjusted risk ratio, 2.17; 95% CI, 1.17-4.04; *P* = .01); however, there was no significant difference during postnatal days 30 to 39 (adjusted risk ratio, 1.26; 95% CI, 0.64-2.49; *P* = .50).

### Secondary Analyses Taking Into Account Days at Risk

**Figure 2.**
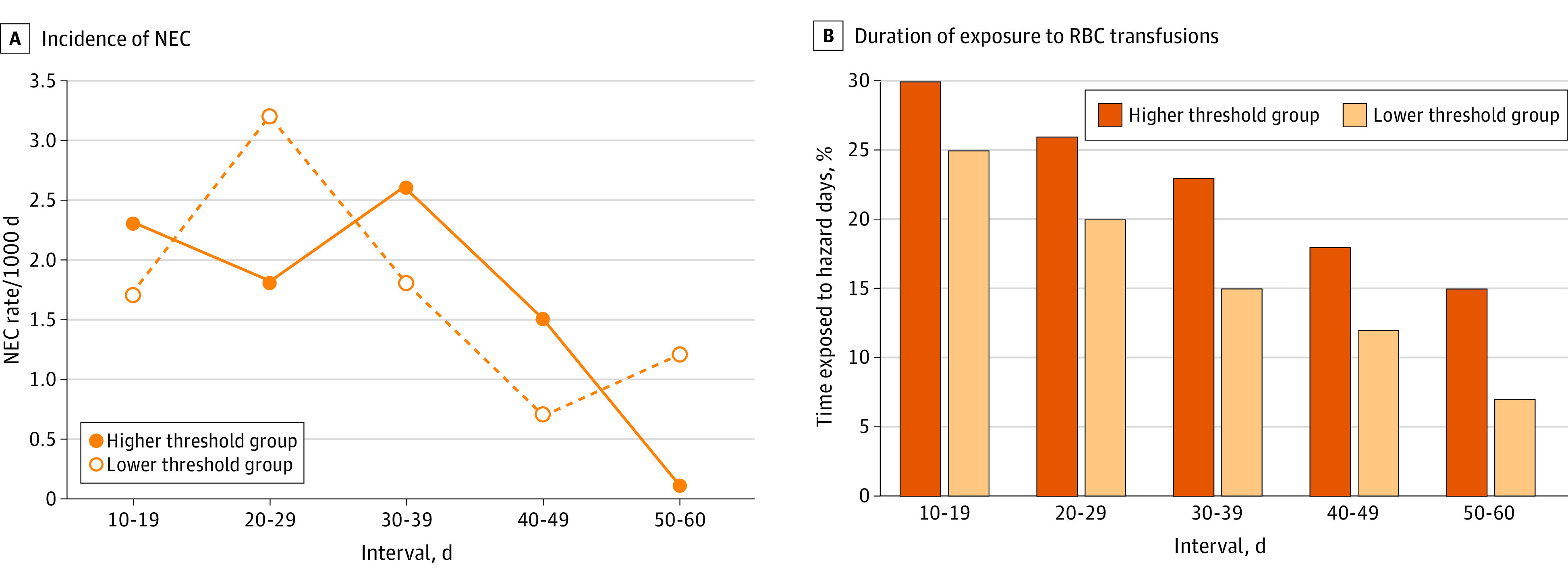
Incidence Rates of Necrotizing Enterocolitis (NEC) and Distribution of Hazard Days for 5 Time Periods From Postnatal Days 10 to 60 by Randomization Group A, Incidence rates of NEC per 1000 days within each 10-day interval, categorized by randomization group. B, Proportion of time within each 10-day interval that infants were exposed to red blood cell (RBC) transfusions, also categorized by randomization group. During the 20- to 29-day interval, infants in the higher hemoglobin transfusion threshold group spent an additional 0.6 days under exposure to RBC transfusions compared with infants in the lower hemoglobin transfusion threshold group and did not exhibit a higher incidence rate of NEC per 1000 days (1.8 vs 3.2; *P* = .09). During the 30- to 39-day interval, infants in the higher hemoglobin transfusion threshold group spent an additional 0.8 days under exposure to RBC transfusions and did not exhibit a higher incidence rate of NEC per 1000 days (2.6 vs 1.8; *P* = .38).

During the specific 20- to 29-day time frame, the incidence rates of NEC per 1000 days were higher, with the lower threshold group exhibiting the highest incidence rates (Figure 2A). Despite this, the 6% difference observed between the 2 threshold groups in the time exposed to hazard days during this specific 10-day interval did not surpass the mean 7% difference seen in other 10-day periods **(**Figure 2B**)**. Further time-to-event analyses focusing solely on hazard or control days during this time frame revealed an incidence rate per 1000 hazard days of 7.92 in the lower threshold group and 4.85 in the higher threshold group (Table 3).

**Table 3.** Time-to-Event Analyses: Incidence Rates of NEC per 1000 Hazard or Control Days for 5 Time Periods From Postnatal Days 10 to 60 by Randomization Group

Outcome by period	Interval and randomization group										Total	
	10-19 d		20-29 d		30-39 d		40-49 d		50-60 d			
	H	L	H	L	H	L	H	L	H	L	H	L
Hazard period												
NEC events	6	4	10	13	8	6	5	4	0	3	29	30
Hazard days	2464	2056	2060	1642	1730	1175	1342	902	1063	556	8659	6331
NEC rate per 1000 hazard days	2.44	1.95	4.85	7.92	4.62	5.11	3.73	4.43	0	5.40	3.35	4.74
Control period												
NEC events	13	10	4	13	12	8	6	1	1	6	36	38
Control days	5738	6282	5816	6351	5907	6514	6046	6562	6114	6690	29 621	32 399
NEC rate per 1000 control days	2.27	1.59	0.69	2.05	2.03	1.23	0.99	0.15	0.16	0.90	1.22	1.17

Abbreviations: H, higher hemoglobin transfusion threshold group; L, lower hemoglobin transfusion threshold group; NEC, necrotizing enterocolitis.

## Discussion

By comparing control and hazard periods, we established that the frequency of NEC events during hazard periods of fixed duration (72 hours) was comparable with the frequency of NEC events during control periods of variable duration. When we compared rates of NEC among infants randomized to the lower threshold group with rates of NEC among infants randomized to the higher threshold group, these rates did not differ between groups. Similarly, baseline and clinical characteristics of infants who had NEC during control periods did not differ from those of infants who had NEC during hazard periods. These comparisons suggest that RBC transfusions are not temporally associated with NEC in ELBW infants with the hemoglobin ranges studied in the TOP trial.

The complexity of establishing temporality in the cause-effect relationship between transient exposure to RBC transfusion and NEC cannot be underestimated. If the exposure status changes intermittently over time, the denominator chosen to report risk is crucial, as risk can be expressed as a function of number of periods at risk, number of days at risk, and number of infants at risk.

In a traditional case-crossover study, only a within-participant comparison of hazard and control periods was performed among patients with the outcome of interest.^18^ Our study uses a denominator containing both within-participant and between-participant comparisons of hazard and control periods among patients with and without the outcome of interest. This type of within- and between-participant comparison accounts for all the days included in the observation period, considers each RBC transfusion event separately, and captures the concept of temporality in a deliberate way. When an RBC transfusion is used as the reference point that defines present time, it becomes much simpler to distinguish the pretransfusion past from the posttransfusion future. Thus, should an RBC transfusion lead to an acute complication such as NEC, the occurrence of NEC will be observed in the immediate posttransfusion period. Because all infants contribute with control periods, the need for covariate adjustments is reduced. Furthermore, by comparing NEC rates during hazard and control periods in stratified analyses according to randomization groups, we minimized the confounding effect of anemia.

Traditional time-to-event analyses using hazard or control days as denominators lack the ability to capture temporality after intermittent exposure to RBC transfusions effectively. When all hazard, posttransfusion days are aggregated to generate the total count of exposure days, as presented in Table 3, days from the remote and immediate past are combined, leading to temporal ambiguity (eg, days from the second week after birth are mingled with those from the fifth or sixth week after birth). Moreover, this approach ignores the predominance of control days at late postnatal ages when NEC rates are low.

By using the total number of days at risk as the denominator within each of the five 10-day intervals, we partially mitigated the issue of mixing days from different time frames. The results derived from these time-to-event analyses suggest that the incidence rate of NEC peaks between postnatal days 20 and 29 among infants with lower hemoglobin values (Figure 2A), especially during hazard days (Table 3). Although these findings differ from the results of the primary analysis, they are not without limitations. The diverse array of analytic approaches used to define the risk of NEC associated with an RBC transfusion^19^ might explain the divergence of results in past studies attempting to determine an association between RBC transfusions and NEC.^4,5,11,20^

The TOP trial used a clinical algorithm to avoid severe anemia that depended on postnatal age and respiratory illness severity (hemoglobin threshold range, 7-10 g/dL). Hence, we recognize that our study may have failed to find an association between severe anemia and NEC after an RBC transfusion. It is possible our results cannot be extrapolated to infants whose hemoglobin values fall beyond the parameters outlined in the TOP trial. Because the trial prevented severe forms of anemia often associated with a higher risk of NEC,^5,20^ it remains possible that a lower hemoglobin threshold could increase this rate due to more severe anemia. As detailed in Table 3, NEC cases were more likely to occur during hazard days of infants in the lower threshold group compared with hazard days of infants in the higher threshold group. This finding warrants a more in-depth examination using larger data sets.

### Limitations

We acknowledge several limitations of our study. First, the study design assumes a comparable baseline risk of NEC among all study participants at the beginning of the observation period. In addition, our outcome evaluation relied on the clinical interpretation of NEC and the current radiographic definition of NEC. Due to surveillance bias, clinicians predisposed to believe that RBC transfusions cause NEC are more likely to observe clinical signs of NEC and order abdominal radiographs during the posttransfusion period. This bias introduces a form of differential misclassification, whereby an increased probability exists of missing NEC cases during the pretransfusion control periods and detecting more NEC cases during the posttransfusion hazard periods. Generally, the onset of symptoms occurs before any radiographic evidence of NEC is present. Moreover, anemia prompting the need for an RBC transfusion could be a sign of NEC.

Another limitation is that we assumed that the first 72 hours after an RBC transfusion would carry the greatest risk for NEC. The precise interval between exposure to RBC transfusions and NEC is unknown.^21^ The same is true for the latent period, the time interval in which a problem exists but has not yet been diagnosed. By performing sensitivity analyses and reanalyzing the data using other definitions of a hazard period (120 hours as opposed to 72 hours), we were able to attenuate these limitations. These supplementary analyses increased the mean duration of hazard periods, made this mean more comparable with the mean duration of control periods, and validated our primary results. Alternative methods, including Bayesian analyses to avoid dichotomous decision-making in our interpretation and multiple-level regression equations, could also be considered to attenuate these limitations.

Not analyzing donor characteristics was the other limitation of the study.^22,23^ The final limitation was the insufficient power to detect the statistical significance of the small differences found between groups, especially in comparisons by randomization groups within each 10-day interval. Some of these 95% CIs are wide, creating uncertainty. Most NEC events were observed within the 20- to 29-day period, which represented the highest risk window for NEC in this secondary analysis. Within this specific high-risk time frame, the lower threshold group exhibited the highest incidence rate of NEC during hazard days, whereas the higher threshold group exhibited the lowest incidence rate of NEC during control days. These results indicate a potential link between severe anemia and the occurrence of NEC during posttransfusion periods. However, our study lacked the necessary statistical power to draw definitive conclusions on this matter.

Prior reviews have yielded conflicting conclusions regarding various feeding practices and the development of NEC. Nonetheless, we await results of the WHEAT trial (Withholding Enteral Feeds Around Packed Red Cell Transfusion to Prevent NEC in Preterm Neonates), which tests the effects of witholding enteral feeding during RBC transfusions on risk reduction of NEC.^24,25^ Our secondary analysis did not delve into the association between enteral feeding during RBC transfusions and NEC.

## Conclusions

This post hoc analysis suggests that, among ELBW infants with hemoglobin ranges defined by the TOP trial, RBC transfusions are not temporally associated with a higher risk of NEC during 72-hour posttransfusion hazard periods. A more in-depth examination of the peak incidence rate of NEC between postnatal days 20 and 29 among infants with lower hemoglobin values is warranted in future studies with larger sample sizes.

## Notes

Supplement 1.
Trial Protocol

Supplement 2. **eFigure 1.** CONSORT Flow Diagram **eFigure 2.** Time-to-Event Plot of 133 Infants Diagnosed With NEC During the Observation Period **eTable.** Stratified Analysis According to Randomization Groups With a 120-h Definition

Supplement 3.
Nonauthor Collaborators

Supplement 4.
Data Sharing Statement
