# Supplementary material for: Timing of Red Blood Cell Transfusions and Occurrence of Necrotizing Enterocolitis: A Secondary Analysis of a Randomized Clinical Trial
Source: JAMA Netw Open. 2024 May 3;7(5):e249643. doi: 10.1001/jamanetworkopen.2024.9643 (PMC11069076; doi:10.1001/jamanetworkopen.2024.9643)
Supplement: Supplement 2. — eFigure 1. CONSORT Flow Diagram eFigure 2. Time-to-Event Plot of 133 Infants Diagnosed With NEC During the Observation Period eTable. Stratified Analysis According to Randomization Groups With a 120-h Definition [file jamanetwopen-e249643-s002.pdf]

# Supplementary Online Content

Salas AA, Gunn, E, Carlo WA, et al. Timing of red blood cell transfusions and occurrence of necrotizing enterocolitis: a secondary analysis of a randomized clinical trial. *JAMA Netw Open.* 2024;7(5):e249643. doi:10.1001/jamanetworkopen.2024.9643

**eFigure 1.** CONSORT Flow Diagram

**eFigure 2.** Time-to-Event Plot of 133 Infants Diagnosed With NEC During the Observation Period

**eTable.** Stratified Analysis According to Randomization Groups With a 120-h Definition

This supplementary material has been provided by the authors to give readers additional information about their work.

© 2024 Salas AA et al. *JAMA Network Open.*

**eFigure 1.** CONSORT Flow Diagram

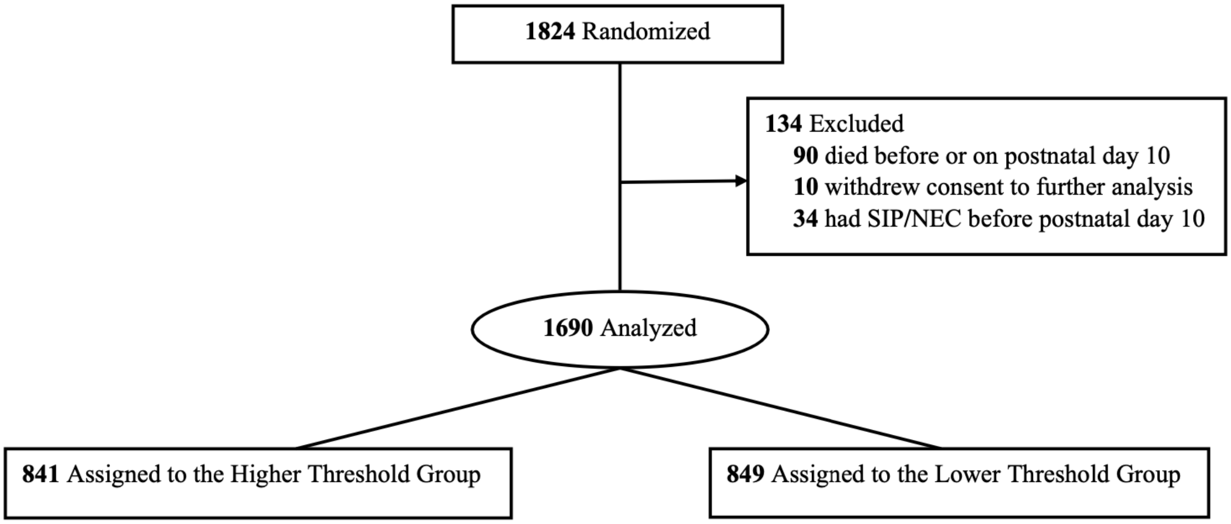


© 2024 Salas AA et al. *JAMA Network Open.*

**eFigure 2.** Time-to-Event Plot of 133 Infants Diagnosed With NEC During the Observation Period

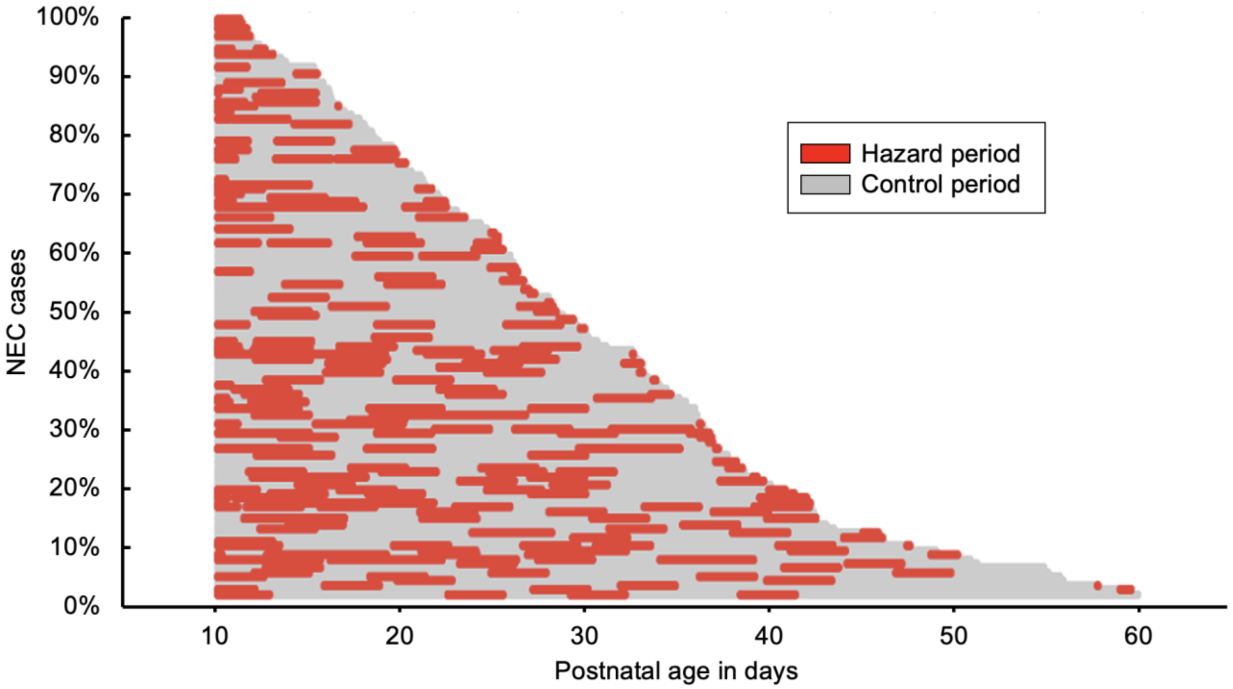


Each horizontal bar in the figure corresponds to an infant diagnosed with NEC. Within each horizontal bar, hazard periods are indicated by red sections, and control periods are represented by gray sections. Using the conceptual model illustrated in Figure 1, we organized NEC cases in an ascending order based on the time of diagnosis.

The x-axis represents postnatal age in days. Late cases, occurring closer to postnatal day 60, are positioned at the bottom of the figure, while early cases, closer to postnatal day 10, are displayed at the top. The y-axis portrays the proportion of NEC cases. Reading the y-axis from bottom to top, we can deduce that approximately 20% of NEC cases occurred between postnatal days 40 and 60, 60% occurred between postnatal day 20 and 39, and the remaining 20% occurred prior to postnatal day 20. This presentation provides a comprehensive visual representation of the distribution of exposure and hazard periods in infants diagnosed with NEC, facilitating a clear understanding of the temporal patterns related to NEC diagnosis.

© 2024 Salas AA et al. *JAMA Network Open.*

**eTable.** Stratified Analysis According to Randomization Groups With a 120-h Definition

| | **High threshold group (Higher hemoglobin)** | | **Low threshold group (Lower hemoglobin)** | |
|---|---|---|---|---|
| | **Hazard periods** (mean duration: 5 days) | **Control periods** (mean duration: 9 days) | **Hazard periods** (mean duration: 5 days) | **Control periods** (mean duration: 12 days) |
| **Number of days** | 13816 | 24464 | 10139 | 28594 |
| **NEC events** | 38 | 27 | 33 | 35 |
| **Number of periods** | 2630 | 2774 | 1962 | 2280 |
| **NEC rate per 1000 periods** | 14.4 | 9.7 | 16.8 | 15.4 |
| **Unadjusted Risk ratio** | 1.47 (95% CI: 0.91 – 2.39) | | 1.10 (95% CI: 0.69 – 1.75) | |
| **Ratio of risk ratios (high threshold: low threshold)** | 1.34 (0.68 – 2.62), p=0.39 | | | |

© 2024 Salas AA et al. *JAMA Network Open.*
