# Supplementary material for: Timing of Red Blood Cell Transfusions and Occurrence of Necrotizing Enterocolitis: A Secondary Analysis of a Randomized Clinical Trial
Source: JAMA Netw Open. 2024 May 3;7(5):e249643. doi: 10.1001/jamanetworkopen.2024.9643 (PMC11069076; doi:10.1001/jamanetworkopen.2024.9643)
Supplement: Supplement 3. — Nonauthor Collaborators [file jamanetwopen-e249643-s003.pdf]

Supplemental Online Content: Nonauthor Collaborators
*First name, last name, and suffix (if applicable) are required and will appear in PubMed.

*Group Name(s): Eunice Kennedy Shriver National Institute of Child Health and Human Development Neonatal Research Network							
*First Name and Middle Initial(s)	*Last Name	*Suffix (eg, Jr, III)	Academic Degrees	Institution	Location (city, state/province, country)	Role or Contribution, eg, chair, principal investigator	Group (if more than 1 Group listed in the byline) and/or Subgroup (eg, Steering Committee)
Richard A.	Polin		MD	Columbia University	New York City, NY, USA	Non-author contributor	
Abbot R.	Laptook		MD	Alpert Medical School of Brown University and Women & Infants Hospital of Rhode Island	Providence, RI, USA	Non-author contributor	
Martin	Keszler		MD	Alpert Medical School of Brown University and Women & Infants Hospital of Rhode Island	Providence, RI, USA	Non-author contributor	
Angelita M.	Hensman		PhD; RNC-NIC	Alpert Medical School of Brown University and Women & Infants Hospital of Rhode Island	Providence, RI, USA	Non-author contributor	
Emily	Little		BSN; RN	Alpert Medical School of Brown University and Women & Infants Hospital of Rhode Island	Providence, RI, USA	Non-author contributor	
Lucille	St. Pierre		BS	Alpert Medical School of Brown University and Women & Infants Hospital of Rhode Island	Providence, RI, USA	Non-author contributor	
Elisa	Vieira		BSN; RN	Alpert Medical School of Brown University and Women & Infants Hospital of Rhode Island	Providence, RI, USA	Non-author contributor	
Michele C.	Walsh		MD; MS	Case Western Reserve University, Rainbow Babies & Children's Hospital	Cleveland, OH, USA	Non-author contributor	
Anna Maria	Hibbs		MD; MSCE	Case Western Reserve University, Rainbow Babies & Children's Hospital	Cleveland, OH, USA	Non-author contributor	
Nancy S.	Newman		RN	Case Western Reserve University, Rainbow Babies & Children's Hospital	Cleveland, OH, USA	Non-author contributor	
Allison H.	Payne		MD; MS	Case Western Reserve University, Rainbow Babies & Children's Hospital	Cleveland, OH, USA	Non-author contributor	
Deanne E.	Wilson-Costello		MD	Case Western Reserve University, Rainbow Babies & Children's Hospital	Cleveland, OH, USA	Non-author contributor	
Bonnie S.	Siner		RN	Case Western Reserve University, Rainbow Babies & Children's Hospital	Cleveland, OH, USA	Non-author contributor	
Monika	Bhola		MD	Case Western Reserve University, Rainbow Babies & Children's Hospital	Cleveland, OH, USA	Non-author contributor	
Gulgun	Yalcinkaya		MD	Case Western Reserve University, Rainbow Babies & Children's Hospital	Cleveland, OH, USA	Non-author contributor	
William E.	Truog		MD	Children's Mercy Hospital	Kansas City, MO, USA	Non-author contributor	
Eugenia K.	Pallotto		MD; MSCE	Children's Mercy Hospital	Kansas City, MO, USA	Non-author contributor	
Howard W.	Kilbride		MD	Children's Mercy Hospital	Kansas City, MO, USA	Non-author contributor	
Cheri	Gauldin		RN; BS; CCRC	Children's Mercy Hospital	Kansas City, MO, USA	Non-author contributor	
Anne	Holmes		RN; MSN; MBA-HCM; CCRC	Children's Mercy Hospital	Kansas City, MO, USA	Non-author contributor	
Kathy	Johnson		RN; CCRC	Children's Mercy Hospital	Kansas City, MO, USA	Non-author contributor	
Allison	Scott		RNC-NIC; BSN; CCRC	Children's Mercy Hospital	Kansas City, MO, USA	Non-author contributor	
Brenda B.	Poindexter		MD; MS	Cincinnati Children's Hospital Medical Center, University Hospital, and Good Samaritan Hospital	Cincinnati, OH, USA	Non-author contributor	
Stephanie L.	Merhar		MD; MS	Cincinnati Children's Hospital Medical Center, University Hospital, and Good Samaritan Hospital	Cincinnati, OH, USA	Non-author contributor	

Supplemental Online Content: Nonauthor Collaborators
*First name, last name, and suffix (if applicable) are required and will appear in PubMed.

*First Name and Middle Initial(s)	*Last Name	*Suffix (eg, Jr, III)	Academic Degrees	Institution	Location (city, state/province, country)	Role or Contribution, eg, chair, principal investigator	Group (if more than 1 Group listed in the byline) and/or Subgroup (eg, Steering Committee)
Kurt	Schibler		MD	Cincinnati Children's Hospital Medical Center, University Hospital, and Good Samaritan Hospital	Cincinnati, OH, USA	Non-author contributor	
Cathy	Grisby		BSN; CCRC	Cincinnati Children's Hospital Medical Center, University Hospital, and Good Samaritan Hospital	Cincinnati, OH, USA	Non-author contributor	
Kristin	Kirker		CRC	Cincinnati Children's Hospital Medical Center, University Hospital, and Good Samaritan Hospital	Cincinnati, OH, USA	Non-author contributor	
Sandra	Wuertz		RN; BSN; CLC	Cincinnati Children's Hospital Medical Center, University Hospital, and Good Samaritan Hospital	Cincinnati, OH, USA	Non-author contributor	
Lenora	Jackson		CRC	Cincinnati Children's Hospital Medical Center, University Hospital, and Good Samaritan Hospital	Cincinnati, OH, USA	Non-author contributor	
Greg	Muthig		BA	Cincinnati Children's Hospital Medical Center, University Hospital, and Good Samaritan Hospital	Cincinnati, OH, USA	Non-author contributor	
Juanita	Dudley		RN; BSN	Cincinnati Children's Hospital Medical Center, University Hospital, and Good Samaritan Hospital	Cincinnati, OH, USA	Non-author contributor	
Robin K.	Whyte		B.Sc; MB; BS	Dalhousie University	Halifax, NS, Canada	Non-author contributor	
C. Michael	Cotten		MD; MHS	Duke University School of Medicine, University Hospital, University of North Carolina, Duke Regional Hospital, and WakeMed Health & Hospitals	Durham, NC, USA	Non-author contributor	
Ronald N.	Goldberg		MD	Duke University School of Medicine, University Hospital, University of North Carolina, Duke Regional Hospital, and WakeMed Health & Hospitals	Durham, NC, USA	Non-author contributor	
Ricki F.	Goldstein		MD	Duke University School of Medicine, University Hospital, University of North Carolina, Duke Regional Hospital, and WakeMed Health & Hospitals	Durham, NC, USA	Non-author contributor	
William F.	Malcolm		MD	Duke University School of Medicine, University Hospital, University of North Carolina, Duke Regional Hospital, and WakeMed Health & Hospitals	Durham, NC, USA	Non-author contributor	
Patricia L.	Ashley		MD	Duke University School of Medicine, University Hospital, University of North Carolina, Duke Regional Hospital, and WakeMed Health & Hospitals	Durham, NC, USA	Non-author contributor	
Deesha	Mago-Shah		MD	Duke University School of Medicine, University Hospital, University of North Carolina, Duke Regional Hospital, and WakeMed Health & Hospitals	Durham, NC, USA	Non-author contributor	
Mollie	Warren		MD	Duke University School of Medicine, University Hospital, University of North Carolina, Duke Regional Hospital, and WakeMed Health & Hospitals	Durham, NC, USA	Non-author contributor	

Supplemental Online Content: Nonauthor Collaborators
*First name, last name, and suffix (if applicable) are required and will appear in PubMed.

*First Name and Middle Initial(s)	*Last Name	*Suffix (eg, Jr, III)	Academic Degrees	Institution	Location (city, state/province, country)	Role or Contribution, eg, chair, principal investigator	Group (if more than 1 Group listed in the byline) and/or Subgroup (eg, Steering Committee)
Joanne	Finkle		RN; JD	Duke University School of Medicine, University Hospital, University of North Carolina, Duke Regional Hospital, and WakeMed Health & Hospitals	Durham, NC, USA	Non-author contributor	
Kimberley A.	Fisher		PhD; FNP-BC; IBCLC	Duke University School of Medicine, University Hospital, University of North Carolina, Duke Regional Hospital, and WakeMed Health & Hospitals	Durham, NC, USA	Non-author contributor	
Kathryn E.	Gustafson		PhD	Duke University School of Medicine, University Hospital, University of North Carolina, Duke Regional Hospital, and WakeMed Health & Hospitals	Durham, NC, USA	Non-author contributor	
Matthew M.	Laughon		MD; MPH	Duke University School of Medicine, University Hospital, University of North Carolina, Duke Regional Hospital, and WakeMed Health & Hospitals	Durham, NC, USA	Non-author contributor	
Carl L.	Bose		MD	Duke University School of Medicine, University Hospital, University of North Carolina, Duke Regional Hospital, and WakeMed Health & Hospitals	Durham, NC, USA	Non-author contributor	
Janice	Bernhardt		MS; RN	Duke University School of Medicine, University Hospital, University of North Carolina, Duke Regional Hospital, and WakeMed Health & Hospitals	Durham, NC, USA	Non-author contributor	
Janice	Wereszczak		CPNP-AC/PC	Duke University School of Medicine, University Hospital, University of North Carolina, Duke Regional Hospital, and WakeMed Health & Hospitals	Durham, NC, USA	Non-author contributor	
Diane	Warner		MD; MPH	Duke University School of Medicine, University Hospital, University of North Carolina, Duke Regional Hospital, and WakeMed Health & Hospitals	Durham, NC, USA	Non-author contributor	
Jennifer	Talbert		MS; RN	Duke University School of Medicine, University Hospital, University of North Carolina, Duke Regional Hospital, and WakeMed Health & Hospitals	Durham, NC, USA	Non-author contributor	
Cindy	Clark		RN	Duke University School of Medicine, University Hospital, University of North Carolina, Duke Regional Hospital, and WakeMed Health & Hospitals	Durham, NC, USA	Non-author contributor	
Stephen D.	Kicklighter		MD	Duke University School of Medicine, University Hospital, University of North Carolina, Duke Regional Hospital, and WakeMed Health & Hospitals	Durham, NC, USA	Non-author contributor	
Alexandra	Bentley		MD	Duke University School of Medicine, University Hospital, University of North Carolina, Duke Regional Hospital, and WakeMed Health & Hospitals	Durham, NC, USA	Non-author contributor	

*First name, last name, and suffix (if applicable) are required and will appear in PubMed.

*First Name and Middle Initial(s)	*Last Name	*Suffix (eg, Jr, III)	Academic Degrees	Institution	Location (city, state/province, country)	Role or Contribution, eg, chair, principal investigator	Group (if more than 1 Group listed in the byline) and/or Subgroup (eg, Steering Committee)
Laura	Edwards		MD	Duke University School of Medicine, University Hospital, University of North Carolina, Duke Regional Hospital, and WakeMed Health & Hospitals	Durham, NC, USA	Non-author contributor	
Ginger	Rhodes-Ryan		ARNP MSN; NNP-BC	Duke University School of Medicine, University Hospital, University of North Carolina, Duke Regional Hospital, and WakeMed Health & Hospitals	Durham, NC, USA	Non-author contributor	
Donna	White		RN-BC; BSN	Duke University School of Medicine, University Hospital, University of North Carolina, Duke Regional Hospital, and WakeMed Health & Hospitals	Durham, NC, USA	Non-author contributor	
Ravi M.	Patel		MD; MSc	Emory University, Children's Healthcare of Atlanta, Grady Memorial Hospital, and Emory University Hospital Midtown	Atlanta, GA, USA	Non-author contributor	
David P.	Carlton		MD	Emory University, Children's Healthcare of Atlanta, Grady Memorial Hospital, and Emory University Hospital Midtown	Atlanta, GA, USA	Non-author contributor	
Barbara J.	Stoll		MD	Emory University, Children's Healthcare of Atlanta, Grady Memorial Hospital, and Emory University Hospital Midtown	Atlanta, GA, USA	Non-author contributor	
Ira	Adams-Chapman		MD	Emory University, Children's Healthcare of Atlanta, Grady Memorial Hospital, and Emory University Hospital Midtown	Atlanta, GA, USA	Non-author contributor	
Yvonne	Loggins		RN	Emory University, Children's Healthcare of Atlanta, Grady Memorial Hospital, and Emory University Hospital Midtown	Atlanta, GA, USA	Non-author contributor	
Ellen C.	Hale		BS; RN; CCRC	Emory University, Children's Healthcare of Atlanta, Grady Memorial Hospital, and Emory University Hospital Midtown	Atlanta, GA, USA	Non-author contributor	
Diane	Bottcher		RN	Emory University, Children's Healthcare of Atlanta, Grady Memorial Hospital, and Emory University Hospital Midtown	Atlanta, GA, USA	Non-author contributor	
Sheena L.	Carter		PhD	Emory University, Children's Healthcare of Atlanta, Grady Memorial Hospital, and Emory University Hospital Midtown	Atlanta, GA, USA	Non-author contributor	
Salathiel	Kendrick-Allwood		MD	Emory University, Children's Healthcare of Atlanta, Grady Memorial Hospital, and Emory University Hospital Midtown	Atlanta, GA, USA	Non-author contributor	
Maureen	Mulligan LaRossa		RN	Emory University, Children's Healthcare of Atlanta, Grady Memorial Hospital, and Emory University Hospital Midtown	Atlanta, GA, USA	Non-author contributor	
Colleen	Mackie		RPT	Emory University, Children's Healthcare of Atlanta, Grady Memorial Hospital, and Emory University Hospital Midtown	Atlanta, GA, USA	Non-author contributor	
Gloria	Smikle		PNP	Emory University, Children's Healthcare of Atlanta, Grady Memorial Hospital, and Emory University Hospital Midtown	Atlanta, GA, USA	Non-author contributor	
Lynn C.	Comerford		NNP	Emory University, Children's Healthcare of Atlanta, Grady Memorial Hospital, and Emory University Hospital Midtown	Atlanta, GA, USA	Non-author contributor	

*First Name and Middle Initial(s)	*Last Name	*Suffix (eg, Jr, III)	Academic Degrees	Institution	Location (city, state/province, country)	Role or Contribution, eg, chair, principal investigator	Group (if more than 1 Group listed in the byline) and/or Subgroup (eg, Steering Committee)
Rosemary D.	Higgins		MD	Eunice Kennedy Shriver National Institute of Child Health and Human Development	Bethesda, MD, USA	Non-author contributor	
Andrew A.	Bremer		MD; PhD	Eunice Kennedy Shriver National Institute of Child Health and Human Development	Bethesda, MD, USA	Non-author contributor	
Stephanie	Wilson Archer		MA	Eunice Kennedy Shriver National Institute of Child Health and Human Development	Bethesda, MD, USA	Non-author contributor	
Gregory M.	Sokol		MD	Indiana University, University Hospital, Methodist Hospital, Riley Hospital for Children, and Wishard Health Services	Indianapolis, IN, USA	Non-author contributor	
Brenda B.	Poindexter		MD; MS	Indiana University, University Hospital, Methodist Hospital, Riley Hospital for Children, and Wishard Health Services	Indianapolis, IN, USA	Non-author contributor	
Heidi	Harmon		MD; MS	Indiana University, University Hospital, Methodist Hospital, Riley Hospital for Children, and Wishard Health Services	Indianapolis, IN, USA	Non-author contributor	
Lu Ann	Papile		MD	Indiana University, University Hospital, Methodist Hospital, Riley Hospital for Children, and Wishard Health Services	Indianapolis, IN, USA	Non-author contributor	
Abbey C.	Hines		PsyD; HSPP	Indiana University, University Hospital, Methodist Hospital, Riley Hospital for Children, and Wishard Health Services	Indianapolis, IN, USA	Non-author contributor	
Dianne E.	Herron		RN; CCRC	Indiana University, University Hospital, Methodist Hospital, Riley Hospital for Children, and Wishard Health Services	Indianapolis, IN, USA	Non-author contributor	
Susan	Gunn		NNP-BC; CCRC	Indiana University, University Hospital, Methodist Hospital, Riley Hospital for Children, and Wishard Health Services	Indianapolis, IN, USA	Non-author contributor	
Lucy	Smiley		CCRC	Indiana University, University Hospital, Methodist Hospital, Riley Hospital for Children, and Wishard Health Services	Indianapolis, IN, USA	Non-author contributor	
Jeff	Joyce		CCRC	Indiana University, University Hospital, Methodist Hospital, Riley Hospital for Children, and Wishard Health Services	Indianapolis, IN, USA	Non-author contributor	
John A. F.	Zupancic		MD; ScD	Harvard Medical School, Beth Israel Deaconess Medical Center	Boston, MA, USA	Non-author contributor	
Kathleen A.	Kennedy		MD; MPH	McGovern Medical School at The University of Texas Health Science Center at Houston, Children's Memorial Hermann Hospital, and Memorial Hermann Southwest Hospital	Houston, TX, USA	Non-author contributor	
Jon E.	Tyson		MD; MPH	McGovern Medical School at The University of Texas Health Science Center at Houston, Children's Memorial Hermann Hospital, and Memorial Hermann Southwest Hospital	Houston, TX, USA	Non-author contributor	
Amir M.	Khan		MD	McGovern Medical School at The University of Texas Health Science Center at Houston, Children's Memorial Hermann Hospital, and Memorial Hermann Southwest Hospital	Houston, TX, USA	Non-author contributor	
Barbara J.	Stoll		MD	McGovern Medical School at The University of Texas Health Science Center at Houston, Children's Memorial Hermann Hospital, and Memorial Hermann Southwest Hospital	Houston, TX, USA	Non-author contributor	

*First name, last name, and suffix (if applicable) are required and will appear in PubMed.

*First Name and Middle Initial(s)	*Last Name	*Suffix (eg, Jr, III)	Academic Degrees	Institution	Location (city, state/province, country)	Role or Contribution, eg, chair, principal investigator	Group (if more than 1 Group listed in the byline) and/or Subgroup (eg, Steering Committee)
Julie	Arldt-McAlister		RN; BSN	McGovern Medical School at The University of Texas Health Science Center at Houston, Children's Memorial Hermann Hospital, and Memorial Hermann Southwest Hospital	Houston, TX, USA	Non-author contributor	
Elizabeth	Eason		MD	McGovern Medical School at The University of Texas Health Science Center at Houston, Children's Memorial Hermann Hospital, and Memorial Hermann Southwest Hospital	Houston, TX, USA	Non-author contributor	
Carmen	Garcia		RN; CCRP	McGovern Medical School at The University of Texas Health Science Center at Houston, Children's Memorial Hermann Hospital, and Memorial Hermann Southwest Hospital	Houston, TX, USA	Non-author contributor	
Donna J.	Hall		RN	McGovern Medical School at The University of Texas Health Science Center at Houston, Children's Memorial Hermann Hospital, and Memorial Hermann Southwest Hospital	Houston, TX, USA	Non-author contributor	
Karen	Martin		RN	McGovern Medical School at The University of Texas Health Science Center at Houston, Children's Memorial Hermann Hospital, and Memorial Hermann Southwest Hospital	Houston, TX, USA	Non-author contributor	
Georgia E.	McDavid		RN	McGovern Medical School at The University of Texas Health Science Center at Houston, Children's Memorial Hermann Hospital, and Memorial Hermann Southwest Hospital	Houston, TX, USA	Non-author contributor	
Shawna L.	Rodgers		RNC-NIC; BSN	McGovern Medical School at The University of Texas Health Science Center at Houston, Children's Memorial Hermann Hospital, and Memorial Hermann Southwest Hospital	Houston, TX, USA	Non-author contributor	
Emily K.	Stephens		RN; BSN	McGovern Medical School at The University of Texas Health Science Center at Houston, Children's Memorial Hermann Hospital, and Memorial Hermann Southwest Hospital	Houston, TX, USA	Non-author contributor	
Sharon L.	Wright		MT (ASCP)	McGovern Medical School at The University of Texas Health Science Center at Houston, Children's Memorial Hermann Hospital, and Memorial Hermann Southwest Hospital	Houston, TX, USA	Non-author contributor	
Traci H.	Mondoro		PhD	National Heart, Lung, and Blood Institute	Bethesda, MD, USA	Non-author contributor	
Catherine	Levy		MHA; BSN; RN	National Heart, Lung, and Blood Institute	Bethesda, MD, USA	Non-author contributor	
Pablo J.	Sánchez		MD	Nationwide Children’s Hospital, Abigail Wexner Research Institute at Nationwide Children’s Hospital, Center for Perinatal Research, The Ohio State University Wexner Medical Center, and Riverside Methodist Hospital	Columbus, OH, USA	Non-author contributor	
Leif D.	Nelin		MD	Nationwide Children’s Hospital, Abigail Wexner Research Institute at Nationwide Children’s Hospital, Center for Perinatal Research, The Ohio State University Wexner Medical Center, and Riverside Methodist Hospital	Columbus, OH, USA	Non-author contributor	

*First Name and Middle Initial(s)	*Last Name	*Suffix (eg, Jr, III)	Academic Degrees	Institution	Location (city, state/province, country)	Role or Contribution, eg, chair, principal investigator	Group (if more than 1 Group listed in the byline) and/or Subgroup (eg, Steering Committee)
Nathalie L.	Maitre		MD; PhD	Nationwide Children's Hospital, Abigail Wexner Research Institute at Nationwide Children's Hospital, Center for Perinatal Research, The Ohio State University Wexner Medical Center, and Riverside Methodist Hospital	Columbus, OH, USA	Non-author contributor	
Ruth B.	Seabrook		MD	Nationwide Children's Hospital, Abigail Wexner Research Institute at Nationwide Children's Hospital, Center for Perinatal Research, The Ohio State University Wexner Medical Center, and Riverside Methodist Hospital	Columbus, OH, USA	Non-author contributor	
Sudarshan R.	Jadcherla		MD	Nationwide Children's Hospital, Abigail Wexner Research Institute at Nationwide Children's Hospital, Center for Perinatal Research, The Ohio State University Wexner Medical Center, and Riverside Methodist Hospital	Columbus, OH, USA	Non-author contributor	
Patricia	Luzader		RN	Nationwide Children's Hospital, Abigail Wexner Research Institute at Nationwide Children's Hospital, Center for Perinatal Research, The Ohio State University Wexner Medical Center, and Riverside Methodist Hospital	Columbus, OH, USA	Non-author contributor	
Christine A.	Fortney		PhD; RN	Nationwide Children's Hospital, Abigail Wexner Research Institute at Nationwide Children's Hospital, Center for Perinatal Research, The Ohio State University Wexner Medical Center, and Riverside Methodist Hospital	Columbus, OH, USA	Non-author contributor	
Julie	Gutentag		RN; BSN	Nationwide Children's Hospital, Abigail Wexner Research Institute at Nationwide Children's Hospital, Center for Perinatal Research, The Ohio State University Wexner Medical Center, and Riverside Methodist Hospital	Columbus, OH, USA	Non-author contributor	
Christopher J.	Timan		MD	Nationwide Children's Hospital, Abigail Wexner Research Institute at Nationwide Children's Hospital, Center for Perinatal Research, The Ohio State University Wexner Medical Center, and Riverside Methodist Hospital	Columbus, OH, USA	Non-author contributor	
Kristi	Small		BS	Nationwide Children's Hospital, Abigail Wexner Research Institute at Nationwide Children's Hospital, Center for Perinatal Research, The Ohio State University Wexner Medical Center, and Riverside Methodist Hospital	Columbus, OH, USA	Non-author contributor	

*First name, last name, and suffix (if applicable) are required and will appear in PubMed.

*First Name and Middle Initial(s)	*Last Name	*Suffix (eg, Jr, III)	Academic Degrees	Institution	Location (city, state/province, country)	Role or Contribution, eg, chair, principal investigator	Group (if more than 1 Group listed in the byline) and/or Subgroup (eg, Steering Committee)
Rox Ann	Sullivan		RN; BSN	Nationwide Children's Hospital, Abigail Wexner Research Institute at Nationwide Children's Hospital, Center for Perinatal Research, The Ohio State University Wexner Medical Center, and Riverside Methodist Hospital	Columbus, OH, USA	Non-author contributor	
Lina	Yoseff-Salameh		MD	Nationwide Children's Hospital, Abigail Wexner Research Institute at Nationwide Children's Hospital, Center for Perinatal Research, The Ohio State University Wexner Medical Center, and Riverside Methodist Hospital	Columbus, OH, USA	Non-author contributor	
Jacqueline	McCool			Nationwide Children's Hospital, Abigail Wexner Research Institute at Nationwide Children's Hospital, Center for Perinatal Research, The Ohio State University Wexner Medical Center, and Riverside Methodist Hospital	Columbus, OH, USA	Non-author contributor	
Melanie	Stein		RRT BBA	Nationwide Children's Hospital, Abigail Wexner Research Institute at Nationwide Children's Hospital, Center for Perinatal Research, The Ohio State University Wexner Medical Center, and Riverside Methodist Hospital	Columbus, OH, USA	Non-author contributor	
Erin	Fearns			Nationwide Children's Hospital, Abigail Wexner Research Institute at Nationwide Children's Hospital, Center for Perinatal Research, The Ohio State University Wexner Medical Center, and Riverside Methodist Hospital	Columbus, OH, USA	Non-author contributor	
Aubrey	Fowler		BS	Nationwide Children's Hospital, Abigail Wexner Research Institute at Nationwide Children's Hospital, Center for Perinatal Research, The Ohio State University Wexner Medical Center, and Riverside Methodist Hospital	Columbus, OH, USA	Non-author contributor	
Jennifer	Grothause		RN; BSN	Nationwide Children's Hospital, Abigail Wexner Research Institute at Nationwide Children's Hospital, Center for Perinatal Research, The Ohio State University Wexner Medical Center, and Riverside Methodist Hospital	Columbus, OH, USA	Non-author contributor	
Stephanie	Burkhardt		BS; MPH	Nationwide Children's Hospital, Abigail Wexner Research Institute at Nationwide Children's Hospital, Center for Perinatal Research, The Ohio State University Wexner Medical Center, and Riverside Methodist Hospital	Columbus, OH, USA	Non-author contributor	

Supplemental Online Content: Nonauthor Collaborators
*First name, last name, and suffix (if applicable) are required and will appear in PubMed.

*First Name and Middle Initial(s)	*Last Name	*Suffix (eg, Jr, III)	Academic Degrees	Institution	Location (city, state/province, country)	Role or Contribution, eg, chair, principal investigator	Group (if more than 1 Group listed in the byline) and/or Subgroup (eg, Steering Committee)
Jessica	Purnell		BS; CCRC	Nationwide Children's Hospital, Abigail Wexner Research Institute at Nationwide Children's Hospital, Center for Perinatal Research, The Ohio State University Wexner Medical Center, and Riverside Methodist Hospital	Columbus, OH, USA	Non-author contributor	
Mary Ann	Nelin		MD	Nationwide Children's Hospital, Abigail Wexner Research Institute at Nationwide Children's Hospital, Center for Perinatal Research, The Ohio State University Wexner Medical Center, and Riverside Methodist Hospital	Columbus, OH, USA	Non-author contributor	
Helen	Carey		PT DHSc PCS	Nationwide Children's Hospital, Abigail Wexner Research Institute at Nationwide Children's Hospital, Center for Perinatal Research, The Ohio State University Wexner Medical Center, and Riverside Methodist Hospital	Columbus, OH, USA	Non-author contributor	
Lindsay	Pietruszewski		PT DPT	Nationwide Children's Hospital, Abigail Wexner Research Institute at Nationwide Children's Hospital, Center for Perinatal Research, The Ohio State University Wexner Medical Center, and Riverside Methodist Hospital	Columbus, OH, USA	Non-author contributor	
Margaret	Sullivan		BS	Nationwide Children's Hospital, Abigail Wexner Research Institute at Nationwide Children's Hospital, Center for Perinatal Research, The Ohio State University Wexner Medical Center, and Riverside Methodist Hospital	Columbus, OH, USA	Non-author contributor	
Julie	Shadd		BS; RD; LD	Nationwide Children's Hospital, Abigail Wexner Research Institute at Nationwide Children's Hospital, Center for Perinatal Research, The Ohio State University Wexner Medical Center, and Riverside Methodist Hospital	Columbus, OH, USA	Non-author contributor	
Jennifer	Notestine		RN	Nationwide Children's Hospital, Abigail Wexner Research Institute at Nationwide Children's Hospital, Center for Perinatal Research, The Ohio State University Wexner Medical Center, and Riverside Methodist Hospital	Columbus, OH, USA	Non-author contributor	
Cole	Hague		BA; MS	Nationwide Children's Hospital, Abigail Wexner Research Institute at Nationwide Children's Hospital, Center for Perinatal Research, The Ohio State University Wexner Medical Center, and Riverside Methodist Hospital	Columbus, OH, USA	Non-author contributor	

*First name, last name, and suffix (if applicable) are required and will appear in PubMed.

*First Name and Middle Initial(s)	*Last Name	*Suffix (eg, Jr, III)	Academic Degrees	Institution	Location (city, state/province, country)	Role or Contribution, eg, chair, principal investigator	Group (if more than 1 Group listed in the byline) and/or Subgroup (eg, Steering Committee)
Erna	Clark		BA	Nationwide Children's Hospital, Abigail Wexner Research Institute at Nationwide Children's Hospital, Center for Perinatal Research, The Ohio State University Wexner Medical Center, and Riverside Methodist Hospital	Columbus, OH, USA	Non-author contributor	
Michelle	Chao		BS	Nationwide Children's Hospital, Abigail Wexner Research Institute at Nationwide Children's Hospital, Center for Perinatal Research, The Ohio State University Wexner Medical Center, and Riverside Methodist Hospital	Columbus, OH, USA	Non-author contributor	
Courtney	Park		RN	Nationwide Children's Hospital, Abigail Wexner Research Institute at Nationwide Children's Hospital, Center for Perinatal Research, The Ohio State University Wexner Medical Center, and Riverside Methodist Hospital	Columbus, OH, USA	Non-author contributor	
Hallie	Baugher		BS; MSN	Nationwide Children's Hospital, Abigail Wexner Research Institute at Nationwide Children's Hospital, Center for Perinatal Research, The Ohio State University Wexner Medical Center, and Riverside Methodist Hospital	Columbus, OH, USA	Non-author contributor	
Demi R.	Beckford		MHS	Nationwide Children's Hospital, Abigail Wexner Research Institute at Nationwide Children's Hospital, Center for Perinatal Research, The Ohio State University Wexner Medical Center, and Riverside Methodist Hospital	Columbus, OH, USA	Non-author contributor	
Bethany	Miller		RN; BSN	Nationwide Children's Hospital, Abigail Wexner Research Institute at Nationwide Children's Hospital, Center for Perinatal Research, The Ohio State University Wexner Medical Center, and Riverside Methodist Hospital	Columbus, OH, USA	Non-author contributor	
Laura	Marzec		MD	Nationwide Children's Hospital, Abigail Wexner Research Institute at Nationwide Children's Hospital, Center for Perinatal Research, The Ohio State University Wexner Medical Center, and Riverside Methodist Hospital	Columbus, OH, USA	Non-author contributor	
Kyrstin	Warnimont		BS	Nationwide Children's Hospital, Abigail Wexner Research Institute at Nationwide Children's Hospital, Center for Perinatal Research, The Ohio State University Wexner Medical Center, and Riverside Methodist Hospital	Columbus, OH, USA	Non-author contributor	
Carla M.	Bann		PhD	RTI International	Research Triangle Park, NC, USA	Non-author contributor	
Marie G.	Gantz		PhD	RTI International	Research Triangle Park, NC, USA	Non-author contributor	
Donald J.	Brambilla		PhD	RTI International	Research Triangle Park, NC, USA	Non-author contributor	

Supplemental Online Content: Nonauthor Collaborators
*First name, last name, and suffix (if applicable) are required and will appear in PubMed.

*First Name and Middle Initial(s)	*Last Name	*Suffix (eg, Jr, III)	Academic Degrees	Institution	Location (city, state/province, country)	Role or Contribution, eg, chair, principal investigator	Group (if more than 1 Group listed in the byline) and/or Subgroup (eg, Steering Committee)
Jamie E.	Newman		PhD; MPH	RTI International	Research Triangle Park, NC, USA	Non-author contributor	
Jenna	Gabrio		MPH	RTI International	Research Triangle Park, NC, USA	Non-author contributor	
Margaret M.	Crawford		BS; CCRP	RTI International	Research Triangle Park, NC, USA	Non-author contributor	
Carolyn M.	Petrie Huitema		MS	RTI International	Research Triangle Park, NC, USA	Non-author contributor	
Jeanette	O'Donnel Auman		BS	RTI International	Research Triangle Park, NC, USA	Non-author contributor	
David	Leblond		BS	RTI International	Research Triangle Park, NC, USA	Non-author contributor	
Annie	vonLehmden		BS	RTI International	Research Triangle Park, NC, USA	Non-author contributor	
Dennis	Wallace		PhD	RTI International	Research Triangle Park, NC, USA	Non-author contributor	
Kristin M.	Zaterka-Baxter		RN; BSN	RTI International	Research Triangle Park, NC, USA	Non-author contributor	
Krisa P.	Van Meurs		MD	Stanford University, El Camino Hospital, and Lucile Packard Children's Hospital	Palo Alto, CA, USA	Non-author contributor	
David K.	Stevenson		MD	Stanford University, El Camino Hospital, and Lucile Packard Children's Hospital	Palo Alto, CA, USA	Non-author contributor	
Susan R.	Hintz		MD; MS	Stanford University, El Camino Hospital, and Lucile Packard Children's Hospital	Palo Alto, CA, USA	Non-author contributor	
M. Bethany	Ball		BSc; CCRC	Stanford University, El Camino Hospital, and Lucile Packard Children's Hospital	Palo Alto, CA, USA	Non-author contributor	
Valerie Y.	Chock		MD; MS	Stanford University, El Camino Hospital, and Lucile Packard Children's Hospital	Palo Alto, CA, USA	Non-author contributor	
Barbara	Bentley		PsyD; MSEd	Stanford University, El Camino Hospital, and Lucile Packard Children's Hospital	Palo Alto, CA, USA	Non-author contributor	
Maria Elena	DeAnda		PhD	Stanford University, El Camino Hospital, and Lucile Packard Children's Hospital	Palo Alto, CA, USA	Non-author contributor	
Anne M.	DeBattista		RN; PNP; PhD	Stanford University, El Camino Hospital, and Lucile Packard Children's Hospital	Palo Alto, CA, USA	Non-author contributor	
Beth	Earhart		PhD	Stanford University, El Camino Hospital, and Lucile Packard Children's Hospital	Palo Alto, CA, USA	Non-author contributor	
Lynne C.	Huffman		MD	Stanford University, El Camino Hospital, and Lucile Packard Children's Hospital	Palo Alto, CA, USA	Non-author contributor	
Casey E.	Krueger		PhD	Stanford University, El Camino Hospital, and Lucile Packard Children's Hospital	Palo Alto, CA, USA	Non-author contributor	
Ryan E.	Lucash		PhD	Stanford University, El Camino Hospital, and Lucile Packard Children's Hospital	Palo Alto, CA, USA	Non-author contributor	
Melinda S.	Proud		RCP	Stanford University, El Camino Hospital, and Lucile Packard Children's Hospital	Palo Alto, CA, USA	Non-author contributor	
Elizabeth N.	Reichert		MA; CCRC	Stanford University, El Camino Hospital, and Lucile Packard Children's Hospital	Palo Alto, CA, USA	Non-author contributor	
Heather	Taylor		PhD	Stanford University, El Camino Hospital, and Lucile Packard Children's Hospital	Palo Alto, CA, USA	Non-author contributor	
Hali E.	Weiss		MD	Stanford University, El Camino Hospital, and Lucile Packard Children's Hospital	Palo Alto, CA, USA	Non-author contributor	
Namasivayam	Ambalavanan		MD	University of Alabama at Birmingham Health System and Children's Hospital of Alabama	Birmingham, AL, USA	Non-author contributor	
Monica V.	Collins		RN; BSN; MaEd	University of Alabama at Birmingham Health System and Children's Hospital of Alabama	Birmingham, AL, USA	Non-author contributor	
Shirley S.	Cosby		RN; BSN	University of Alabama at Birmingham Health System and Children's Hospital of Alabama	Birmingham, AL, USA	Non-author contributor	
Myriam Peralta	Carcelen		MD; MPH	University of Alabama at Birmingham Health System and Children's Hospital of Alabama	Birmingham, AL, USA	Non-author contributor	
Fred J.	Biasini		PhD	University of Alabama at Birmingham Health System and Children's Hospital of Alabama	Birmingham, AL, USA	Non-author contributor	

*First name, last name, and suffix (if applicable) are required and will appear in PubMed.

*First Name and Middle Initial(s)	*Last Name	*Suffix (eg, Jr, III)	Academic Degrees	Institution	Location (city, state/province, country)	Role or Contribution, eg, chair, principal investigator	Group (if more than 1 Group listed in the byline) and/or Subgroup (eg, Steering Committee)
Kristen C.	Johnston		MSN; CRNP	University of Alabama at Birmingham Health System and Children's Hospital of Alabama	Birmingham, AL, USA	Non-author contributor	
Mary Beth	Moses		PT; MS; PCS	University of Alabama at Birmingham Health System and Children's Hospital of Alabama	Birmingham, AL, USA	Non-author contributor	
Tara E.	McNair		RN; BSN	University of Alabama at Birmingham Health System and Children's Hospital of Alabama	Birmingham, AL, USA	Non-author contributor	
Vivien A.	Phillips		RN; BSN	University of Alabama at Birmingham Health System and Children's Hospital of Alabama	Birmingham, AL, USA	Non-author contributor	
Richard V.	Rector		PhD	University of Alabama at Birmingham Health System and Children's Hospital of Alabama	Birmingham, AL, USA	Non-author contributor	
Sally	Whitley		MA; OTR-L FAOT	University of Alabama at Birmingham Health System and Children's Hospital of Alabama	Birmingham, AL, USA	Non-author contributor	
Kristy A.	Domnanovich		PhD	University of Alabama at Birmingham Health System and Children's Hospital of Alabama	Birmingham, AL, USA	Non-author contributor	
Sheree	York Chapman		PT DPT PCS	University of Alabama at Birmingham Health System and Children's Hospital of Alabama	Birmingham, AL, USA	Non-author contributor	
Uday	Devaskar		MD	University of California - Los Angeles, Mattel Children's Hospital, Santa Monica Hospital, Los Robles Hospital and Medical Center, and Olive View Medical Center	Los Angeles, CA, USA	Non-author contributor	
Meena	Garg		MD	University of California - Los Angeles, Mattel Children's Hospital, Santa Monica Hospital, Los Robles Hospital and Medical Center, and Olive View Medical Center	Los Angeles, CA, USA	Non-author contributor	
Isabell B.	Purdy		PhD; CPNP	University of California - Los Angeles, Mattel Children's Hospital, Santa Monica Hospital, Los Robles Hospital and Medical Center, and Olive View Medical Center	Los Angeles, CA, USA	Non-author contributor	
Teresa	Chanlaw		MPH	University of California - Los Angeles, Mattel Children's Hospital, Santa Monica Hospital, Los Robles Hospital and Medical Center, and Olive View Medical Center	Los Angeles, CA, USA	Non-author contributor	
Rachel	Geller		RN; BSN	University of California - Los Angeles, Mattel Children's Hospital, Santa Monica Hospital, Los Robles Hospital and Medical Center, and Olive View Medical Center	Los Angeles, CA, USA	Non-author contributor	
Tarah T.	Colaizy		MD; MPH	University of Iowa and Sanford Health	Iowa City, IA, USA	Non-author contributor	
Jane E.	Brumbaugh		MD	University of Iowa and Sanford Health	Iowa City, IA, USA	Non-author contributor	
John A.	Widness		MD	University of Iowa and Sanford Health	Iowa City, IA, USA	Non-author contributor	
Heidi M.	Harmon		MD; MS	University of Iowa and Sanford Health	Iowa City, IA, USA	Non-author contributor	
Karen J.	Johnson		RN; BSN	University of Iowa and Sanford Health	Iowa City, IA, USA	Non-author contributor	
Jacky R.	Walker		RN	University of Iowa and Sanford Health	Iowa City, IA, USA	Non-author contributor	
Claire A.	Goeke		DNP; ARNP	University of Iowa and Sanford Health	Iowa City, IA, USA	Non-author contributor	
Diane L.	Eastman		RN; CPNP; MA	University of Iowa and Sanford Health	Iowa City, IA, USA	Non-author contributor	
Kristi L.	Watterberg		MD	University of New Mexico Health Sciences Center	Albuquerque, NM, USA	Non-author contributor	
Robin K.	Ohls		MD	University of New Mexico Health Sciences Center	Albuquerque, NM, USA	Non-author contributor	
Conra	Backstrom Lacy		RN	University of New Mexico Health Sciences Center	Albuquerque, NM, USA	Non-author contributor	

Supplemental Online Content: Nonauthor Collaborators
*First name, last name, and suffix (if applicable) are required and will appear in PubMed.

*First Name and Middle Initial(s)	*Last Name	*Suffix (eg, Jr, III)	Academic Degrees	Institution	Location (city, state/province, country)	Role or Contribution, eg, chair, principal investigator	Group (if more than 1 Group listed in the byline) and/or Subgroup (eg, Steering Committee)
Janell	Fuller		MD	University of New Mexico Health Sciences Center	Albuquerque, NM, USA	Non-author contributor	
Mary	Hanson		RN; BSN	University of New Mexico Health Sciences Center	Albuquerque, NM, USA	Non-author contributor	
Carol	Hartenberger		BSN; MPH	University of New Mexico Health Sciences Center	Albuquerque, NM, USA	Non-author contributor	
Elizabeth	Kuan		RN; BSN	University of New Mexico Health Sciences Center	Albuquerque, NM, USA	Non-author contributor	
Jean R.	Lowe		PhD	University of New Mexico Health Sciences Center	Albuquerque, NM, USA	Non-author contributor	
Sandra	Sundquist Beauman		MSN; RNC	University of New Mexico Health Sciences Center	Albuquerque, NM, USA	Non-author contributor	
Barbara	Schmidt		MD; MSc	University of Pennsylvania, Hospital of the University of Pennsylvania, Pennsylvania Hospital, and Children's Hospital of Philadelphia	Philadelphia, PA, USA	Non-author contributor	
Sara B.	DeMauro		MD; MSCE	University of Pennsylvania, Hospital of the University of Pennsylvania, Pennsylvania Hospital, and Children's Hospital of Philadelphia	Philadelphia, PA, USA	Non-author contributor	
Soraya	Abbasi		MD	University of Pennsylvania, Hospital of the University of Pennsylvania, Pennsylvania Hospital, and Children's Hospital of Philadelphia	Philadelphia, PA, USA	Non-author contributor	
Toni	Mancini		RN; BSN; CCRC	University of Pennsylvania, Hospital of the University of Pennsylvania, Pennsylvania Hospital, and Children's Hospital of Philadelphia	Philadelphia, PA, USA	Non-author contributor	
Dana M.	Cucinotta		RN	University of Pennsylvania, Hospital of the University of Pennsylvania, Pennsylvania Hospital, and Children's Hospital of Philadelphia	Philadelphia, PA, USA	Non-author contributor	
Jonathan	Snyder		RN; BSN	University of Pennsylvania, Hospital of the University of Pennsylvania, Pennsylvania Hospital, and Children's Hospital of Philadelphia	Philadelphia, PA, USA	Non-author contributor	
Aasma S.	Chaudhary		BS; RRT	University of Pennsylvania, Hospital of the University of Pennsylvania, Pennsylvania Hospital, and Children's Hospital of Philadelphia	Philadelphia, PA, USA	Non-author contributor	
Carl T.	D'Angio		MD	University of Rochester Medical Center, Golisano Children's Hospital, and the University of Buffalo Women's and Children's Hospital of Buffalo	Rochester, NY, USA	Non-author contributor	
Ronnie	Guillet		MD; PhD	University of Rochester Medical Center, Golisano Children's Hospital, and the University of Buffalo Women's and Children's Hospital of Buffalo	Rochester, NY, USA	Non-author contributor	

Supplemental Online Content: Nonauthor Collaborators
*First name, last name, and suffix (if applicable) are required and will appear in PubMed.

*First Name and Middle Initial(s)	*Last Name	*Suffix (eg, Jr, III)	Academic Degrees	Institution	Location (city, state/province, country)	Role or Contribution, eg, chair, principal investigator	Group (if more than 1 Group listed in the byline) and/or Subgroup (eg, Steering Committee)
Melissa F.	Carmen		MD	University of Rochester Medical Center, Golisano Children's Hospital, and the University of Buffalo Women's and Children's Hospital of Buffalo	Rochester, NY, USA	Non-author contributor	
Gary J.	Myers		MD	University of Rochester Medical Center, Golisano Children's Hospital, and the University of Buffalo Women's and Children's Hospital of Buffalo	Rochester, NY, USA	Non-author contributor	
Kyle	Binion		BS	University of Rochester Medical Center, Golisano Children's Hospital, and the University of Buffalo Women's and Children's Hospital of Buffalo	Rochester, NY, USA	Non-author contributor	
Caitlin	Fallone		MA	University of Rochester Medical Center, Golisano Children's Hospital, and the University of Buffalo Women's and Children's Hospital of Buffalo	Rochester, NY, USA	Non-author contributor	
Osman	Farooq		MD	University of Rochester Medical Center, Golisano Children's Hospital, and the University of Buffalo Women's and Children's Hospital of Buffalo	Rochester, NY, USA	Non-author contributor	
Julianne	Hunn		MSHCM	University of Rochester Medical Center, Golisano Children's Hospital, and the University of Buffalo Women's and Children's Hospital of Buffalo	Rochester, NY, USA	Non-author contributor	
Rosemary L.	Jensen			University of Rochester Medical Center, Golisano Children's Hospital, and the University of Buffalo Women's and Children's Hospital of Buffalo	Rochester, NY, USA	Non-author contributor	
Joan	Merzbach		LMSW	University of Rochester Medical Center, Golisano Children's Hospital, and the University of Buffalo Women's and Children's Hospital of Buffalo	Rochester, NY, USA	Non-author contributor	
Constance	Orme			University of Rochester Medical Center, Golisano Children's Hospital, and the University of Buffalo Women's and Children's Hospital of Buffalo	Rochester, NY, USA	Non-author contributor	
Ann Marie	Scorsone		MS; CCRC	University of Rochester Medical Center, Golisano Children's Hospital, and the University of Buffalo Women's and Children's Hospital of Buffalo	Rochester, NY, USA	Non-author contributor	
Holly I. M.	Wadkins			University of Rochester Medical Center, Golisano Children's Hospital, and the University of Buffalo Women's and Children's Hospital of Buffalo	Rochester, NY, USA	Non-author contributor	
Kelley	Yost		PhD	University of Rochester Medical Center, Golisano Children's Hospital, and the University of Buffalo Women's and Children's Hospital of Buffalo	Rochester, NY, USA	Non-author contributor	

Supplemental Online Content: Nonauthor Collaborators
*First name, last name, and suffix (if applicable) are required and will appear in PubMed.

*First Name and Middle Initial(s)	*Last Name	*Suffix (eg, Jr, III)	Academic Degrees	Institution	Location (city, state/province, country)	Role or Contribution, eg, chair, principal investigator	Group (if more than 1 Group listed in the byline) and/or Subgroup (eg, Steering Committee)
Satyan	Lakshminrusimha		MD	University of Rochester Medical Center, Golisano Children's Hospital, and the University of Buffalo Women's and Children's Hospital of Buffalo	Rochester, NY, USA	Non-author contributor	
Anne Marie	Reynolds		MD	University of Rochester Medical Center, Golisano Children's Hospital, and the University of Buffalo Women's and Children's Hospital of Buffalo	Rochester, NY, USA	Non-author contributor	
Stephanie	Guilford		BS	University of Rochester Medical Center, Golisano Children's Hospital, and the University of Buffalo Women's and Children's Hospital of Buffalo	Rochester, NY, USA	Non-author contributor	
Michelle E.	Hartley-McAndrews		MD	University of Rochester Medical Center, Golisano Children's Hospital, and the University of Buffalo Women's and Children's Hospital of Buffalo	Rochester, NY, USA	Non-author contributor	
Ashley	Williams		MSEd	University of Rochester Medical Center, Golisano Children's Hospital, and the University of Buffalo Women's and Children's Hospital of Buffalo	Rochester, NY, USA	Non-author contributor	
William	Zorn		PhD	University of Rochester Medical Center, Golisano Children's Hospital, and the University of Buffalo Women's and Children's Hospital of Buffalo	Rochester, NY, USA	Non-author contributor	
Mary	Rowan		RN	University of Rochester Medical Center, Golisano Children's Hospital, and the University of Buffalo Women's and Children's Hospital of Buffalo	Rochester, NY, USA	Non-author contributor	
Dee	Maffett		RN	University of Rochester Medical Center, Golisano Children's Hospital, and the University of Buffalo Women's and Children's Hospital of Buffalo	Rochester, NY, USA	Non-author contributor	
Diane	Prinzing			University of Rochester Medical Center, Golisano Children's Hospital, and the University of Buffalo Women's and Children's Hospital of Buffalo	Rochester, NY, USA	Non-author contributor	
Melissa	Bowman		RN	University of Rochester Medical Center, Golisano Children's Hospital, and the University of Buffalo Women's and Children's Hospital of Buffalo	Rochester, NY, USA	Non-author contributor	
Michael	Sacilowski		MAT CCRC	University of Rochester Medical Center, Golisano Children's Hospital, and the University of Buffalo Women's and Children's Hospital of Buffalo	Rochester, NY, USA	Non-author contributor	
Daisy	Rochesz		BS; MHA	University of Rochester Medical Center, Golisano Children's Hospital, and the University of Buffalo Women's and Children's Hospital of Buffalo	Rochester, NY, USA	Non-author contributor	
Myra H.	Wyckoff		MD	University of Texas Southwestern Medical Center, Parkland Health & Hospital System, and Children's Medical Center Dallas	Dallas, TX, USA	Non-author contributor	

*First name, last name, and suffix (if applicable) are required and will appear in PubMed.

*First Name and Middle Initial(s)	*Last Name	*Suffix (eg, Jr, III)	Academic Degrees	Institution	Location (city, state/province, country)	Role or Contribution, eg, chair, principal investigator	Group (if more than 1 Group listed in the byline) and/or Subgroup (eg, Steering Committee)
Luc P.	Brion		MD	University of Texas Southwestern Medical Center, Parkland Health & Hospital System, and Children's Medical Center Dallas	Dallas, TX, USA	Non-author contributor	
Roy J.	Heyne		MD	University of Texas Southwestern Medical Center, Parkland Health & Hospital System, and Children's Medical Center Dallas	Dallas, TX, USA	Non-author contributor	
Diana M.	Vasil		MSN; BSN; RNC-NIC	University of Texas Southwestern Medical Center, Parkland Health & Hospital System, and Children's Medical Center Dallas	Dallas, TX, USA	Non-author contributor	
Sally S.	Adams		MS; RN; CPNP	University of Texas Southwestern Medical Center, Parkland Health & Hospital System, and Children's Medical Center Dallas	Dallas, TX, USA	Non-author contributor	
Maria M.	De Leon		RN; BSN	University of Texas Southwestern Medical Center, Parkland Health & Hospital System, and Children's Medical Center Dallas	Dallas, TX, USA	Non-author contributor	
Frances	Eubanks		RN; BSN	University of Texas Southwestern Medical Center, Parkland Health & Hospital System, and Children's Medical Center Dallas	Dallas, TX, USA	Non-author contributor	
Alicia	Guzman			University of Texas Southwestern Medical Center, Parkland Health & Hospital System, and Children's Medical Center Dallas	Dallas, TX, USA	Non-author contributor	
Elizabeth	Heyne		PsyD; PA-C	University of Texas Southwestern Medical Center, Parkland Health & Hospital System, and Children's Medical Center Dallas	Dallas, TX, USA	Non-author contributor	
Lizette E.	Lee		RN	University of Texas Southwestern Medical Center, Parkland Health & Hospital System, and Children's Medical Center Dallas	Dallas, TX, USA	Non-author contributor	
Linda A.	Madden		BSN; RN; CPNP	University of Texas Southwestern Medical Center, Parkland Health & Hospital System, and Children's Medical Center Dallas	Dallas, TX, USA	Non-author contributor	
Lara	Pavageau		MD	University of Texas Southwestern Medical Center, Parkland Health & Hospital System, and Children's Medical Center Dallas	Dallas, TX, USA	Non-author contributor	
Pollieanna	Sepulveda		RN	University of Texas Southwestern Medical Center, Parkland Health & Hospital System, and Children's Medical Center Dallas	Dallas, TX, USA	Non-author contributor	
Cathy	Twell Boatman		MS; CIMI	University of Texas Southwestern Medical Center, Parkland Health & Hospital System, and Children's Medical Center Dallas	Dallas, TX, USA	Non-author contributor	
Kristine	Tolentino-Plata		MS	University of Texas Southwestern Medical Center, Parkland Health & Hospital System, and Children's Medical Center Dallas	Dallas, TX, USA	Non-author contributor	
Bradley A.	Yoder		MD	University of Utah Medical Center, Intermountain Medical Center, McKay-Dee Hospital, Utah Valley Hospital, and Primary Children's Hospital	Salt Lake City, UT, USA	Non-author contributor	
Mariana	Baserga		MD; MSCI	University of Utah Medical Center, Intermountain Medical Center, McKay-Dee Hospital, Utah Valley Hospital, and Primary Children's Hospital	Salt Lake City, UT, USA	Non-author contributor	

Supplemental Online Content: Nonauthor Collaborators
*First name, last name, and suffix (if applicable) are required and will appear in PubMed.

*First Name and Middle Initial(s)	*Last Name	*Suffix (eg, Jr, III)	Academic Degrees	Institution	Location (city, state/province, country)	Role or Contribution, eg, chair, principal investigator	Group (if more than 1 Group listed in the byline) and/or Subgroup (eg, Steering Committee)
Stephen D.	Minton		MD	University of Utah Medical Center, Intermountain Medical Center, McKay-Dee Hospital, Utah Valley Hospital, and Primary Children's Hospital	Salt Lake City, UT, USA	Non-author contributor	
Mark J.	Sheffield		MD	University of Utah Medical Center, Intermountain Medical Center, McKay-Dee Hospital, Utah Valley Hospital, and Primary Children's Hospital	Salt Lake City, UT, USA	Non-author contributor	
Carrie A.	Rau		RN; BSN; CCRC	University of Utah Medical Center, Intermountain Medical Center, McKay-Dee Hospital, Utah Valley Hospital, and Primary Children's Hospital	Salt Lake City, UT, USA	Non-author contributor	
Jill	Burnett		RNC; BSN	University of Utah Medical Center, Intermountain Medical Center, McKay-Dee Hospital, Utah Valley Hospital, and Primary Children's Hospital	Salt Lake City, UT, USA	Non-author contributor	
Susan	Christensen		RN	University of Utah Medical Center, Intermountain Medical Center, McKay-Dee Hospital, Utah Valley Hospital, and Primary Children's Hospital	Salt Lake City, UT, USA	Non-author contributor	
Laura	Cole Bledsoe		RN	University of Utah Medical Center, Intermountain Medical Center, McKay-Dee Hospital, Utah Valley Hospital, and Primary Children's Hospital	Salt Lake City, UT, USA	Non-author contributor	
Jennifer O.	Elmont		RN; BSN	University of Utah Medical Center, Intermountain Medical Center, McKay-Dee Hospital, Utah Valley Hospital, and Primary Children's Hospital	Salt Lake City, UT, USA	Non-author contributor	
Trisha	Marchant		RN	University of Utah Medical Center, Intermountain Medical Center, McKay-Dee Hospital, Utah Valley Hospital, and Primary Children's Hospital	Salt Lake City, UT, USA	Non-author contributor	
Earl	Maxson		RN; CCRN	University of Utah Medical Center, Intermountain Medical Center, McKay-Dee Hospital, Utah Valley Hospital, and Primary Children's Hospital	Salt Lake City, UT, USA	Non-author contributor	
Kandace M.	McGrath			University of Utah Medical Center, Intermountain Medical Center, McKay-Dee Hospital, Utah Valley Hospital, and Primary Children's Hospital	Salt Lake City, UT, USA	Non-author contributor	
Hena G.	Mickelsen		BA	University of Utah Medical Center, Intermountain Medical Center, McKay-Dee Hospital, Utah Valley Hospital, and Primary Children's Hospital	Salt Lake City, UT, USA	Non-author contributor	
D. Melody	Parry		RN; BSN	University of Utah Medical Center, Intermountain Medical Center, McKay-Dee Hospital, Utah Valley Hospital, and Primary Children's Hospital	Salt Lake City, UT, USA	Non-author contributor	

*First name, last name, and suffix (if applicable) are required and will appear in PubMed.

*First Name and Middle Initial(s)	*Last Name	*Suffix (eg, Jr, III)	Academic Degrees	Institution	Location (city, state/province, country)	Role or Contribution, eg, chair, principal investigator	Group (if more than 1 Group listed in the byline) and/or Subgroup (eg, Steering Committee)
Brixen A.	Reich		MSN; RNC; CCRC	University of Utah Medical Center, Intermountain Medical Center, McKay-Dee Hospital, Utah Valley Hospital, and Primary Children's Hospital	Salt Lake City, UT, USA	Non-author contributor	
Susan T.	Schaefer		RN; BSN; RRT	University of Utah Medical Center, Intermountain Medical Center, McKay-Dee Hospital, Utah Valley Hospital, and Primary Children's Hospital	Salt Lake City, UT, USA	Non-author contributor	
Kimberlee	Weaver-Lewis		RN; MS	University of Utah Medical Center, Intermountain Medical Center, McKay-Dee Hospital, Utah Valley Hospital, and Primary Children's Hospital	Salt Lake City, UT, USA	Non-author contributor	
Kathryn D.	Woodbury		RN; BSN	University of Utah Medical Center, Intermountain Medical Center, McKay-Dee Hospital, Utah Valley Hospital, and Primary Children's Hospital	Salt Lake City, UT, USA	Non-author contributor	
Seetha	Shankaran		MD	Wayne State University, Hutzel Women's Hospital, Children's Hospital of Michigan, and the University of Michigan C.S. Mott Children's Hospital	Detroit, MI, USA	Non-author contributor	
Beena G.	Sood		MD; MS	Wayne State University, Hutzel Women's Hospital, Children's Hospital of Michigan, and the University of Michigan C.S. Mott Children's Hospital	Detroit, MI, USA	Non-author contributor	
Sanjay	Chawla		MD	Wayne State University, Hutzel Women's Hospital, Children's Hospital of Michigan, and the University of Michigan C.S. Mott Children's Hospital	Detroit, MI, USA	Non-author contributor	
Rebecca	Bara		RN; BSN	Wayne State University, Hutzel Women's Hospital, Children's Hospital of Michigan, and the University of Michigan C.S. Mott Children's Hospital	Detroit, MI, USA	Non-author contributor	
Prashant	Agarwal		MD	Wayne State University, Hutzel Women's Hospital, Children's Hospital of Michigan, and the University of Michigan C.S. Mott Children's Hospital	Detroit, MI, USA	Non-author contributor	
Monika	Bajaj		MD	Wayne State University, Hutzel Women's Hospital, Children's Hospital of Michigan, and the University of Michigan C.S. Mott Children's Hospital	Detroit, MI, USA	Non-author contributor	
Kirsten	Childs		RN; BSN	Wayne State University, Hutzel Women's Hospital, Children's Hospital of Michigan, and the University of Michigan C.S. Mott Children's Hospital	Detroit, MI, USA	Non-author contributor	
Melissa	February		MD	Wayne State University, Hutzel Women's Hospital, Children's Hospital of Michigan, and the University of Michigan C.S. Mott Children's Hospital	Detroit, MI, USA	Non-author contributor	

Supplemental Online Content: Nonauthor Collaborators
*First name, last name, and suffix (if applicable) are required and will appear in PubMed.

*First Name and Middle Initial(s)	*Last Name	*Suffix (eg, Jr, III)	Academic Degrees	Institution	Location (city, state/province, country)	Role or Contribution, eg, chair, principal investigator	Group (if more than 1 Group listed in the byline) and/or Subgroup (eg, Steering Committee)
Laura	Goldston		MA	Wayne State University, Hutzel Women's Hospital, Children's Hospital of Michigan, and the University of Michigan C.S. Mott Children's Hospital	Detroit, MI, USA	Non-author contributor	
Mary E.	Johnson		RN; BSN	Wayne State University, Hutzel Women's Hospital, Children's Hospital of Michigan, and the University of Michigan C.S. Mott Children's Hospital	Detroit, MI, USA	Non-author contributor	
Girija	Natarajan		MD	Wayne State University, Hutzel Women's Hospital, Children's Hospital of Michigan, and the University of Michigan C.S. Mott Children's Hospital	Detroit, MI, USA	Non-author contributor	
Bogdan	Panaitescu		MD; PhD	Wayne State University, Hutzel Women's Hospital, Children's Hospital of Michigan, and the University of Michigan C.S. Mott Children's Hospital	Detroit, MI, USA	Non-author contributor	
Eunice	Woldt		RN; MSN	Wayne State University, Hutzel Women's Hospital, Children's Hospital of Michigan, and the University of Michigan C.S. Mott Children's Hospital	Detroit, MI, USA	Non-author contributor	
John	Barks		MD	Wayne State University, Hutzel Women's Hospital, Children's Hospital of Michigan, and the University of Michigan C.S. Mott Children's Hospital	Detroit, MI, USA	Non-author contributor	
Martha	Carlson		MD	Wayne State University, Hutzel Women's Hospital, Children's Hospital of Michigan, and the University of Michigan C.S. Mott Children's Hospital	Detroit, MI, USA	Non-author contributor	
Diane F.	White		RRT; CCRP	Wayne State University, Hutzel Women's Hospital, Children's Hospital of Michigan, and the University of Michigan C.S. Mott Children's Hospital	Detroit, MI, USA	Non-author contributor	
						Non-author contributor	
