# Supplementary material for: Timing of Red Blood Cell Transfusions and Occurrence of Necrotizing Enterocolitis: A Secondary Analysis of a Randomized Clinical Trial
Source: JAMA Netw Open. 2024 May 3;7(5):e249643. doi: 10.1001/jamanetworkopen.2024.9643 (PMC11069076; doi:10.1001/jamanetworkopen.2024.9643)
Supplement: Supplement 4. — Data Sharing Statement [file jamanetwopen-e249643-s004.pdf]

Data Sharing Statement

Salas. Timing of Red Blood Cell Transfusions and Occurrence of Necrotizing Enterocolitis. *JAMA Netw Open*. Published May 03, 2024. doi:10.1001/jamanetworkopen.2024.9643

Data
Data available: Yes
Data types: Deidentified participant data
How to access data: De-identified individual participant data will be made available upon publication to researchers who provide a methodologically sound proposal for use in achieving the goals of the approved proposal. Data reported in this paper is planned for release to the NHLBI Biospecimen and Data Repository (DASH) at https://biolincc.nhlbi.nih.gov/home/.
When available: With publication

Supporting Documents
Document types: None

Additional Information
Who can access the data: researchers whose proposed use of the data has been approved
Types of analyses: for a specified purpose
Mechanisms of data availability: with a signed data access agreement
